# From symmetry breaking to symmetry swapping: is Kasha's rule violated in multibranched phenyleneethynylenes?†

**DOI:** 10.1039/d2sc05206g

**Published:** 2023-01-13

**Authors:** K. Swathi, Meleppatt Sujith, P. S. Divya, Merin Varghese P, Andrea Delledonne, D. K. Andrea Phan Huu, Francesco Di Maiolo, Francesca Terenziani, Andrea Lapini, Anna Painelli, Cristina Sissa, K. George Thomas

**Affiliations:** a Dipartimento di Scienze Chimiche, della Vita e della Sostenibilità Ambientale, Università di Parma Parco Area delle Scienze 17A 43124 Parma Italy cristina.sissa@unipr.it; b School of Chemistry, Indian Institute of Science Education and Research Thiruvananthapuram (IISER TVM) Vithura Thiruvananthapuram 695 551 India kgt@iisertvm.ac.in

## Abstract

The phenomenon of excited-state symmetry breaking is often observed in multipolar molecular systems, significantly affecting their photophysical and charge separation behavior. As a result of this phenomenon, the electronic excitation is partially localized in one of the molecular branches. However, the intrinsic structural and electronic factors that regulate excited-state symmetry breaking in multibranched systems have hardly been investigated. Herein, we explore these aspects by adopting a joint experimental and theoretical investigation for a class of phenyleneethynylenes, one of the most widely used molecular building blocks for optoelectronic applications. The large Stokes shifts observed for highly symmetric phenyleneethynylenes are explained by the presence of low-lying dark states, as also established by two-photon absorption measurements and TDDFT calculations. In spite of the presence of low-lying dark states, these systems show an intense fluorescence in striking contrast to Kasha's rule. This intriguing behavior is explained in terms of a novel phenomenon, dubbed “symmetry swapping” that describes the inversion of the energy order of excited states, *i.e.*, the swapping of excited states occurring as a consequence of symmetry breaking. Thus, symmetry swapping explains quite naturally the observation of an intense fluorescence emission in molecular systems whose lowest vertical excited state is a dark state. In short, symmetry swapping is observed in highly symmetric molecules having multiple degenerate or quasi-degenerate excited states that are prone to symmetry breaking.

## Abstract 2

Highly symmetric multibranched phenyleneethynylenes exhibit intense fluorescence despite the presence of low-lying dark states. The inversion of the energy order of excited states is explained in terms of a novel phenomenon dubbed “symmetry swapping”.

## Graphical abstract

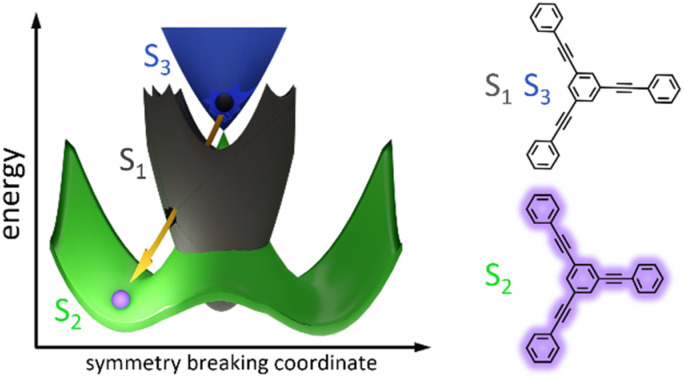

## Introduction

Kasha's rule, an empirical founding principle in molecular spectroscopy, states that fluorescence can only appreciably stem in molecules from the lowest-energy excited state having the same spin multiplicity as the ground state. As a consequence, to observe fluorescence, the lowest excited-state should be a bright state, *i.e.*, it should have a sizable transition dipole moment from the ground state.^1–3^ The rationale behind this phenomenological rule is simple: after excitation, a molecule quickly relaxes to the lowest excited singlet (for simplicity, we consider closed-shell molecules with a singlet ground state) so that fluorescence can be observed from this state, often called Kasha's state. However, to observe spontaneous emission, the fluorescence probability must be large enough to overcome other relaxation processes such as non-radiative decay and intersystem crossing. Kasha's rule explains why J-aggregates are strongly fluorescent while H-aggregates are dark,^4–6^ and it allows fluorescent polymers to be distinguished from non-fluorescent ones based on the symmetry of the excited states, as dictated by electron-correlation and vibronic coupling.^7^ Nonetheless, Kasha's rule is not an exact theorem: even formally non-fluorescent systems may show very weak fluorescence due to vibronic coupling, as in the well-known case of H-aggregates where a weak and red-shifted fluorescence is observed.^8–12^ More impressive deviations from Kasha's rule are observed in the rare systems where dual fluorescence occurs, originating from an anomalously long-lived high-energy singlet state as well as from Kasha's state.^13–17^ In this work, we report a joint experimental and theoretical investigation of highly symmetric multibranched phenyleneethynylenes characterized by a dark Kasha's state, but showing an intense fluorescence. This seeming violation of Kasha's rule is the result of a symmetry-breaking phenomenon that lowers the energy of a bright excited singlet below the dark Kasha's state. Herein we dub this swapping of excited states induced by symmetry-breaking as symmetry swapping. Symmetry breaking is a powerful concept that emerged in the field of condensed matter physics^18^ to describe systems represented by a perfectly symmetric Hamiltonian that collapse into a state with reduced symmetry. Of course, the global symmetry is regained by the presence of several equivalent broken-symmetry states that the system may choose to collapse in. Indeed, multistability is a signature of symmetry breaking. In chemical systems, one of the first examples of symmetry breaking can be traced back to mixed valence complexes, wherein two equivalent metallic centers share a few electrons.^19,20^ In spite of the equivalence of the two metals, if the electronic charge is not equally distributed, a broken-symmetry state is observed. The concept of symmetry breaking was explicitly introduced in the chemical community in a work addressing the anomalous fluorescence solvatochromism in nominally symmetric two-branched charge-transfer dyes.^21^ Spontaneous symmetry breaking in the ground state of long cyanine dyes has also been discussed^22–24^ and, more recently, symmetry lowering induced by external perturbations (counterions) was reported.^25^ In multibranched molecules having formally equivalent branches, symmetry breaking implies a geometrical distortion leading to inequivalent branches in the excited states, as recognized and thoroughly investigated, from both experimental and theoretical perspectives.^26–35^ Symmetry breaking also plays a crucial role in the generation of charge separation in multichromophoric assemblies.^36–42^ In multibranched multipolar chromophores, whose low-lying excitations are dominated by charge transfer degrees of freedom, symmetry breaking is driven by polar solvation, as theoretically predicted^21,26^ and experimentally verified.^29,43,44^ In these multipolar systems, having formally equivalent branches, symmetry breaking implies charge-localization in one branch, with a concomitant geometrical distortion, so that in the relaxed excited state the molecular branches are no more equivalent.

**Scheme 1:**
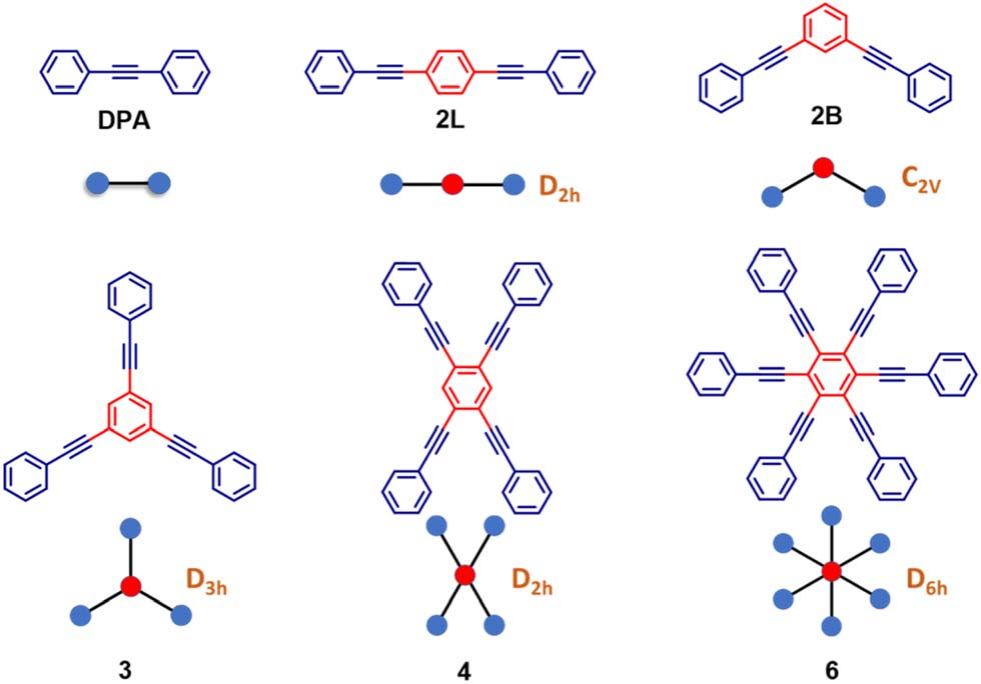
Molecular structures of the phenyleneethynylene derivatives along with their ‘ball and stick’ representation which is used in the subsequent sections. In brown, the symmetry group of the symmetric structure is given (except DPA, the reference compound). PEs are labeled as 2L, 2B, 3, 4, and 6 with numbers 2, 3, 4 and 6 denoting the number of phenylacetylene arms; L and B denote linear and bent geometries, respectively.

Herein, we present the dramatic influence of excited-state symmetry breaking on the photophysics of a family of multibranched molecules (Scheme 1) based on phenyleneethynylenes (PEs). In these molecules charge transfer degrees of freedom are not relevant, due to the lack of electron-donor or acceptor groups. Accordingly, polar solvation plays a marginal role, as demonstrated experimentally in this manuscript. These rigid linear π-conjugated molecular systems show however intriguing physical properties:^45–53^ (i) the rod-like molecular structure of PE is resistant to isomerization and (ii) the cylindrical nature of the carbon–carbon triple bonds maintains the π-electron conjugation at any degree of rotation of the phenyl rings. By exploiting these properties, PE-based motifs with branched phenylacetylene have been extensively exploited for the design of molecular materials. For instance, PE-based molecular systems have been explored for surface engineering, as elements in optoelectronic systems such as in molecular electronics and for the design of donor–acceptor systems.^54–59^ However, a systematic understanding of the effect of structural branching and molecular symmetry on the photophysics of this class of molecules is still lacking. In this regard, multibranched systems having two, three, four and six equivalent phenylacetylene branches, arranged to share the same central benzene unit, have been synthesized as shown in Scheme 1. The molecules are labeled as 2L, 2B, 3, 4, and 6 where the numbers refer to the number of arms, while L and B denote linear and bent geometries, respectively. The presence of an increasing number of branches arranged in different geometries, with different symmetries and degrees of conjugation, offers a perfect playground to understand excited-state symmetry breaking in these systems wherein the role of solvent polarity is marginal due to the absence of electron donating/accepting groups. The high density of excited states in these systems leads to intriguing photophysics and to a variety of distinctively different properties. Specifically, the investigated family is comprised of molecules (Scheme 1) displaying a classical symmetry-preserving behavior as well as excited-state symmetry breaking, with a special case exhibiting the novel symmetry swapping phenomenon.

## Results and discussion

**Scheme 2:**
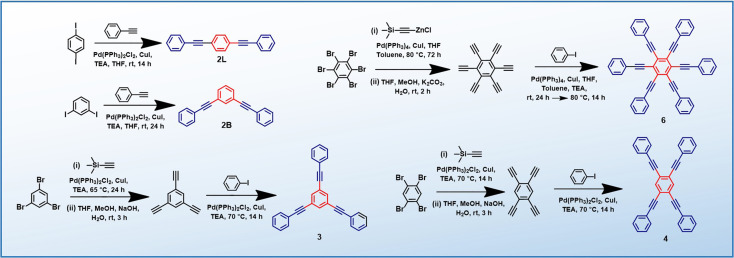
Synthesis of PEs (2L, 2B, 3, 4 and 6).

PEs 2L, 2B, 3 and 4 are synthesized *via* the Heck–Cassar–Sonogashira–Hagihara cross-coupling reaction, while a tandem Negishi–Sonogashira cross-coupling protocol^60^ is adopted for the synthesis of hexaethynylbenzene derivative 6 (Scheme 2). Details of the synthesis and characterization are provided in the ESI.†

### Establishing low-energy dark states in PEs

**Fig. 1:**
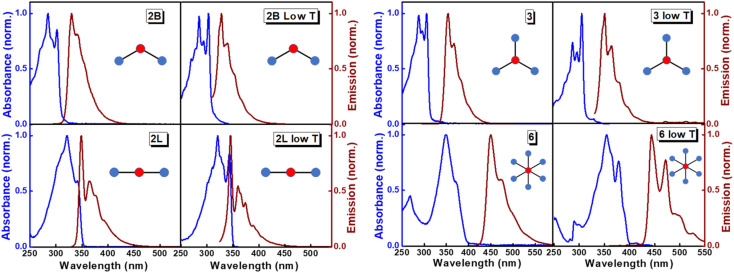
Absorption/excitation (blue lines; absorption spectra were measured at room temperature, while excitation spectra were measured at low temperature) and emission spectra (brown lines) of PEs. For each compound, the left column shows spectra measured in CHCl_3_ at room temperature and the right column shows results in glassy matrices (propylene glycol at 190 K for 2B, 2L and 3; 2-methyl-THF at 77 K for 6).

**Fig. 2:**
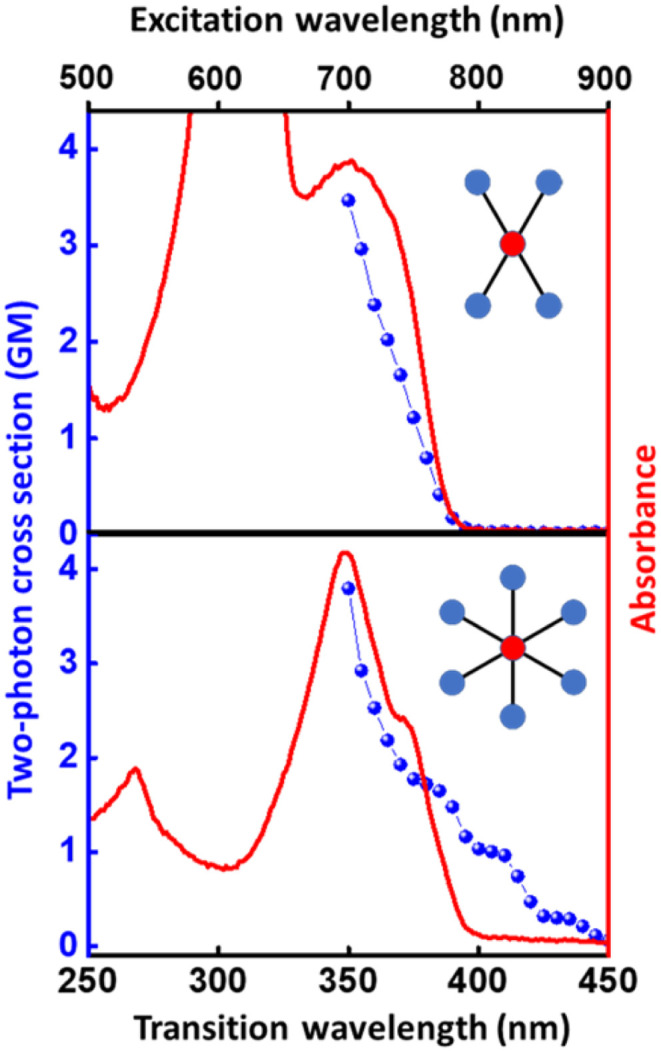
Two-photon absorption spectra (blue dots) of 4 (top) and 6 (bottom) in CHCl_3_. One-photon absorption is shown (continuous red line, arbitrary scale) for comparison. Details about the two-photon absorption measurement are presented in the Experimental section.

**Fig. 3:**
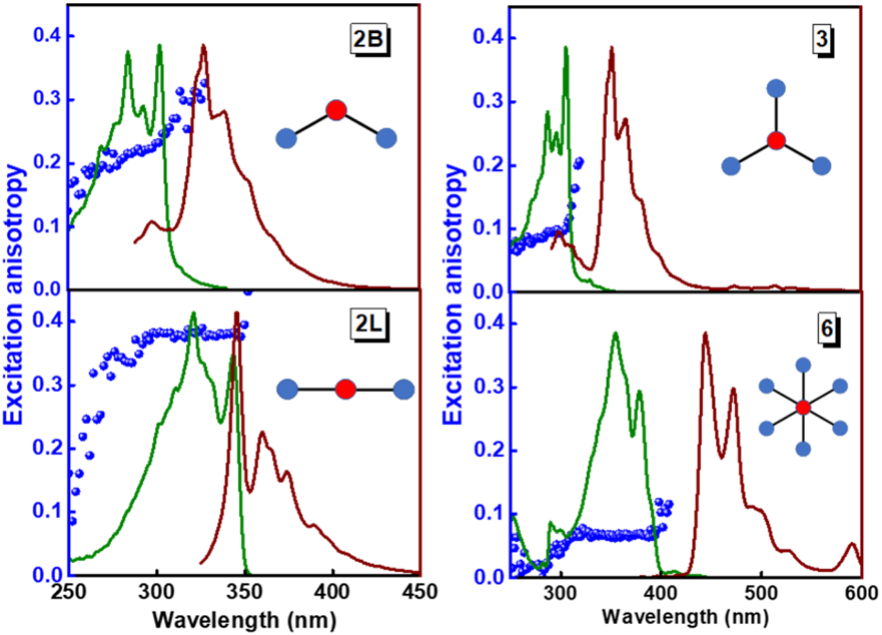
Excitation (green lines), emission (brown lines) and excitation fluorescence anisotropy (blue spheres) of 2B, 2L, and 3 (in propylene glycol at 190 K) and of 6 (in 2MeTHF at 77 K). Excitation wavelengths for anisotropy experiments: 345 nm (2B); 365 nm (2L); 365 nm (3); 450 nm (6). Fluorescence anisotropy spectra of DPA and 4 are reported in Fig. S4.†

The structurally similar PEs in Scheme 1 show contrasting photophysical properties. The UV-vis absorption and fluorescence emission and excitation spectra of 2L, 2B, 3 and 6 dissolved in liquid CHCl_3_ and in glassy matrices are displayed in Fig. 1 (DPA and 4 in Fig. S1†) and relevant data are summarized in Tables 1 and S1.† Of particular interest are the strikingly different Stokes shifts, measured as the energy difference between the 0–0 vibronic transitions in absorption and emission, observed for different compounds. Specifically, data obtained in glassy solvent, reported in Table S2,† are not affected by the solvent relaxation and therefore give direct information on the molecular relaxation upon excitation. The Stokes shift is negligible for both DPA and 2L, and very small for 4, while being sizable for 2B, 3 and 6 (Fig. 1 and S1; Table S2†). These results are in line with the Stokes shifts observed in CHCl_3_ under ambient conditions (Fig. 1 and Table 1), and confirm the marginal role of solvent polarity in these systems, as further supported by the spectra collected in a non-polar solvent, cyclohexane (Fig. S2†). Finally, we notice that 2B, 3 and 6 are moderately fluorescent (*ϕ*_f_ = 0.15–0.32) whereas 2L is highly fluorescent (*ϕ*_f_ = 0.94). An obvious question now arises – why do the Stokes shifts of these PEs display such a large difference? Quite interestingly, the absorption spectra of 3 and 6 show a tiny band located on the red side of the most intense peak, suggesting the presence of low-energy dark states acquiring weak intensity *via* vibronic coupling (Fig. 1 and S3†). Two photon absorption is a convenient and unambiguous technique to locate dark states. We therefore measured two-photon absorption spectra with the two-photon excited fluorescence (TPEF) technique. In our experimental setup (*vide infra*), excitation can be tuned in the 700–1300 nm region so that TPEF data could only be collected for 4 and 6. Fig. 2 shows the two-photon excitation spectra as a function of the transition wavelength (*i.e.*, half the excitation wavelength). Interestingly, two distinct bands are observed in the two-photon excitation spectrum of 6 in the 400–450 nm spectral region, confirming the presence of states that are dark in linear absorption. In contrast, the two-photon excitation spectrum of 4 does not show any signature ascribable to low-energy dark states. These experimental results confirm that the lowest singlet excited states of 6 are optically dark, in striking contrast with the observed sizable fluorescence quantum yield. The presence of multiple excited states very close in energy in 2B, 3 and 6 is further confirmed by fluorescence anisotropy experiments (Fig. 3 and S4†) that were carried out in glassy solvent (to avoid rotational diffusion). PE molecules are further classified into three groups based on the anisotropy spectra presented in Fig. 3: (i) DPA and 2L, (ii) 3 and 6 and (iii) 2B and 4. Excitation anisotropy of DPA and 2L is close to the limiting value of 0.4, and is approximately flat within the absorption band. A marginal wavelength dependence is observed for DPA, which is ascribed to the presence of other excited states at high energy. Molecules 3 and 6 have a flat excitation anisotropy spectrum, but with a distinctively low value of ∼0.05–0.1, suggesting the presence of multiple degenerate excited states (see the ESI† for further discussion). The red-edge effect^61^ is responsible for the abrupt increase of anisotropy in the red edge of the absorption of 3 and marginally in 6. Interestingly, moving from higher to lower wavelength, the excitation anisotropy of 4 and 2B varies from 0.2 to 0 and from 0.3 to 0.2, respectively. The variation of excitation anisotropy in the spectral region of interest is a clear indication of the presence of multiple excited states.

**Table 1:** Absorption and fluorescence properties of PEs in CHCl_3_

PE	*λ*_abs_ a [nm]	*λ*_em_ b [nm]	Stokes shift^c^ [cm^−1^]	Molar extinction coefficient (M^−1^ cm^−1^)	Quantum yield^d^ (*ϕ*_f_)	Lifetime^e^ (ns)
2L	340	349	759	3.42 × 10^4^	0.94 ± 0.01	0.6
2B	302	330	2810	4.58 × 10^4^	0.15 ± 0.03	3.1
3	306	354	4431	10.79 × 10^4^	0.19 ± 0.02	7.8
6	370	450	4805	6.38 × 10^4^	0.32 ± 0.02	20.0

a First vibronic shoulder observed in the spectrum in CHCl_3_.

b First vibronic band of the fluorescence spectrum in CHCl_3_.

c The Stokes shift is estimated in CHCl_3_ at room temperature.

d Fluorescence quantum yields are determined using the relative method, with *p*-terphenyl in cyclohexane as the standard (*ϕ*_f_ = 0.93) for 2L, 2B and 3 with excitation wavelength at 290 nm, and anthracene in cyclohexane as the standard (*ϕ*_f_ = 0.36) for 4 and 6 with excitation wavelength at 355 nm.

e Excitation wavelengths: 314 nm for 2B and 3; 340 nm for 2L and 6. The *χ*^2^ values of the lifetime fittings are in the 0.9–1.2 range.

In an effort to shed light on the intriguing photophysics of these systems, we performed time-resolved transient absorption measurements. Unfortunately, with our experimental setup (see the Experimental section) the pump region does not extend below 350 nm, hindering the measurement on 2B and 3. We therefore investigated molecules 2L and 6 (results in Fig. S5†) as representatives of molecules that show negligible and large Stokes shifts, respectively. The stimulated emission of 6 is observed at 450 nm, and an excited state absorption appears at ∼600 nm. The stimulated emission of 2L falls outside the accessible spectral window, and we only detect the excited state absorption at 620 nm. The decay of the excited state absorption signal of 2L is very fast being almost completed in the first 1.5 ns. In contrast, the signal at 600 nm observed for 6 survives much longer. These results are in line with the different fluorescence decays measured for the two dyes, amounting to 0.6 ns and 20 ns, respectively (Table 1).

### Vertical transitions from the optimized ground state

Having experimentally established the presence of low-lying dark excited states in 3 and 6, we performed TDDFT calculations on the PE structures to obtain deeper insight into their ground and excited-state properties. Ground-state geometries are obtained at the DFT level of theory (CAM-B3LYP functional and 6-31G* basis set; more details in the Experimental section). All calculations are run in the gas phase, since, as confirmed by experimental data (Fig. 1 and S2†), in these molecules the solvent polarity has marginal effects. On the other hand, continuum solvent approaches, commonly adopted to account for solvation, may lead to uncontrolled results, particularly in systems with several excited states that are close in energy and with different characteristics.^62–64^Tables 2 and S3† list absorption energies, oscillator strengths and transition dipoles calculated at the ground-state optimized geometry. All multibranched systems in Scheme 1 show symmetric structures in the ground state, with equivalent branches (Fig. S6†). The calculated vertical transition energies compare well with the experiment. Interestingly, the linear 2L molecule has a single bright transition at low energy, polarized along the main molecular axis, while the corresponding bent molecule 2B shows two almost degenerate bright transitions, polarized along mutually perpendicular directions. Computational results confirm the experimental observation that the lowest-energy excited states are dark for both 3 and 6. Specifically, the first excited-state of 3, and the first and second excited states of 6 have negligible oscillator strength (Table 2). The dark states of either 3 or 6 involve excitations between two pairs of degenerate orbitals, as discussed in detail in the ESI (Section S3.1, Table S5 and Scheme S6†) where we compare the frontier orbitals of 3 and 6 to the frontier orbitals of the DPA molecule. Results in Table S4† point to degenerate HOMO/HOMO−1 and LUMO/LUMO+1 for both 3 and 6, in line with the high symmetry of the two molecules, since both *D*_3h_ and *D*_6h_ groups support doubly degenerate representations.

**Table 2:** Selected TDDFT results at the optimized ground state geometry^a^

Compound	Transition number	Transition wavelength (nm)	Oscillator strength
2L	1	313	1.91
2B	1	275	1.66
	2	271	0.36
3	1	286	0.00
	2	277	1.61
	3	277	1.61
	4	270	0.00
6	1	363	0.00
	2	361	0.00
	3	329	1.88
	4	329	1.88

a Additional data (transition dipole moments, orbital contributions, *etc.*) are reported in Table S3 of the ESI.

### Symmetry breaking and symmetry swapping

**Fig. 4:**
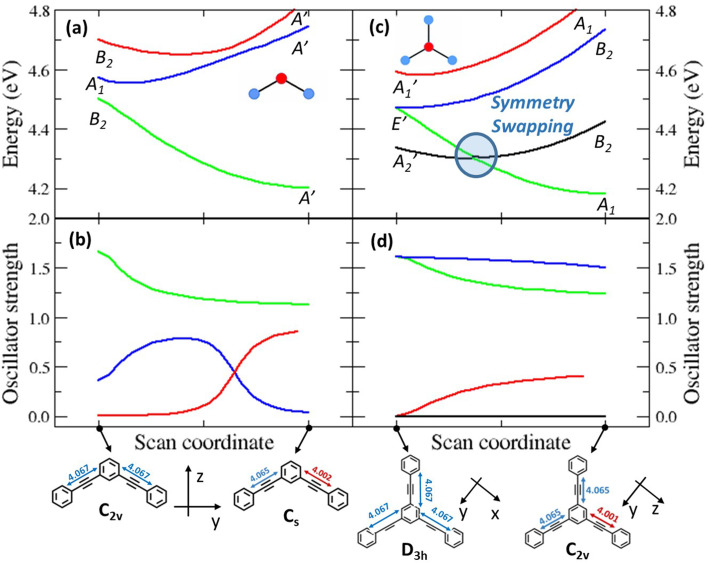
Results of scan calculations performed on molecules 2B, where symmetry breaking occurs, and molecule 3, where a symmetry swapping occurs. The scan is performed by defining an effective coordinate connecting the ground state geometry, *x*_gs_, to the excited state geometry, *x*_es_. Intermediate geometries are obtained moving the atoms from *x*_gs_ along the displacement vector *d* = *x*_es_ − *x*_gs_. A TD-DFT calculation was performed at each step to obtain excited state energies and transition dipole moments. Top panels show the potential energy curves of the lowest excited states, and their symmetry labels. Bottom panels report the oscillator strengths of the same excited states. The start and end points of the calculation are reported on the *x*-axis, corresponding to the optimized ground state geometry and the optimized broken-symmetry excited state geometry, respectively. The symmetry group is reported for each structure, as well as the cartesian reference systems. Bond lengths are reported in Å.

To investigate the geometrical rearrangement in the excited state and obtain information about symmetry breaking, we have optimized the excited states of the PE structures at the TDDFT level of theory (CAM-B3LYP functional, 6-31G* basis set). Results for the PEs showing a large Stokes shift, 2B, 3 and 6, are listed in Table 3, and those for the molecules showing a small Stokes shift, DPA, 2L and 4, are presented in Table S6.† The optimization of the lowest excited-state of 2B leads to a broken-symmetry geometry, with nonequivalent bonds in the two molecular arms (Fig. 4 and S7†) so that the molecular symmetry lowers from *C*_2v_ to *C*_s_. Fig. 4a and b show how the energy and oscillator strength of the three lowest excited states of 2B vary when the system is driven from the ground state geometry to the excited state geometry *via* a continuous variation of the coordinate (see the Experimental section for technical details about the calculation). In the ground state geometry (*C*_2v_ group) the first and third excited states (polarized along the long molecular axis, *y*) transform as B_2_, and the second excited state (polarized along *z*, the short molecular axis) transforms as A_1_. As soon as we move along the symmetry breaking coordinate, the molecular symmetry lowers to *C*_s_ and the three states all transform as A′. The two lowest energy states are very close in energy and their mixing is responsible for the lowering of the energy of the first excited state, driving the symmetry-breaking. Both states are optically bright in the ground state geometry, even if they are polarized along orthogonal directions: their mixing of course changes the direction of polarization. Quite interestingly, an avoided crossing between the second and third excited states is responsible for the redistribution of oscillator strength between the two states. We notice that a specularly reversed broken symmetry geometry is obtained from the optimization of the second excited-state (Table 3 and Fig. S7†).

**Table 3:** TDDFT results obtained from the optimized excited-state geometries of molecules 2B, 3 and 6

Compound	Optimized excited-state^a^	Transition wavelength^b^	Oscillator strength
2B	1	319	1.13
	2	319	1.13
3	1	305	0.00
	2	321	1.24
	3	321	1.24
6	1	391	0.00
	2	394	0.36
	3	349	1.85
	4	349	1.85

a The number of the excited-states is reported in the order they are at the ground state equilibrium geometry.

b Computed transition wavelength from the optimized excited-state to the ground state.

The situation is much more interesting for molecule 3. In the ground state equilibrium geometry, the three molecular branches are equivalent and the molecule belongs to the *D*_3h_ group. As discussed in the ESI,† the first and fourth excited states (A_2_′ and A_1_′ symmetry, respectively) are dark states, while the second and third excited states are degenerate (E′ symmetry) and are optically bright, having a large transition dipole moment from the ground state. The E′ states are indeed responsible for the intense absorption of 3 at 306 nm, while the dark A_2_′ state is responsible for the weak (vibronically allowed, top right panel in Fig. 1 and S3†) transition at ≈330 nm, as also supported by fluorescence excitation anisotropy data (Fig. 3). The optimized first excited state stays dark as the molecule maintains the *D*_3h_ symmetry (Table 3 and Fig. S7†). This result does not explain the large fluorescence quantum yield of 3. Nonetheless, upon optimization, the second and third excited states of 3 undergo symmetry breaking: both optimizations converge towards two equivalent geometries where one molecular arm is different from the other two (Table 3 and Fig. S7†), reducing the molecular symmetry to *C*_2v_. These relaxed states are bright and, most importantly, their energy is lower than the energy of the optimized first excited state. Fig. 4c and d show the evolution of energies and of oscillator strengths with the symmetry-breaking coordinate of the four lowest excited states of 3. When moving away from the ground state geometry, the lowest excited state evolves into a B_2_ state in the *C*_2v_ group and the fourth excited state into A_1_, while the two degenerate E′ states in *D*_3h_ become non-degenerate and evolve into A_1_ and B_2_ states in *C*_2v_. As long as two states have the same symmetry, they can mix. Specifically, the mixing between the two states with A_1_ symmetry lowers the energy of one state below the energy of the B_2_ state, as to produce a symmetry swapping, *i.e.*, a swapping of excited-states induced by symmetry breaking*.*

The B_1_ state, while optically allowed by symmetry, maintains a negligible oscillator strength that cannot explain the intense fluorescence of 3. Indeed, the fluorescent state is one of the E′ states that evolves into a low energy A_1_ state in the broken symmetry geometry. For the sake of completeness, Fig. S8† shows the analogous geometry scan, performed along the effective coordinate that connects the optimized ground-state geometry to the optimized geometry of the first (dark) excited state, confirming again that upon optimization of the A_2_′ state, symmetry is conserved and the state has higher energy than the A_1_ state in the broken symmetry geometry. Fig. S9 and S10† show the scans for molecules 4 and 6, respectively. We stress that the potential energy surfaces in Fig. 4 and S8–S10† give information about the energy of the different states at the different geometries, but they cannot provide any information about the actual relaxation path followed by the molecule.

In summary, in the ground state geometry, as relevant to absorption processes, the lowest excited-state is a dark state so that 3 should not be a fluorescent molecule according to Kasha's rule. However, when the molecule is excited to the bright doubly degenerate E′ states, it relaxes to a broken-symmetry geometry. In this relaxed geometry, the bright state has lower energy than the dark state (Fig. 4c), conclusively explaining the large fluorescence quantum yield of 3 without violating Kasha's rule. We designate this new phenomenon of symmetry-induced excited-state swapping as symmetry swapping.

The situation is more complex for 6, due to the presence of several excited states with similar energies and the possibility of having different stable conformers either in the ground or in the excited states, making the geometry optimization difficult. However, as for 3, the first excited-state of 6 stays dark and symmetric upon geometry optimization. Instead, the second excited-state of 6, a dark state at the ground state geometry, and almost degenerate with the first excited-state (Table 2), breaks the symmetry upon relaxation (two arms become non-equivalent with respect to the other four arms – Fig. S7 and S10†) and becomes bright. The two lowest relaxed excited states of 6 are very close in energy, so that the bright and dark states are thermally populated, justifying the observed emission (Fig. S10†). Overall, all molecules showing a large Stokes shift, 2B, 3 and 6, undergo symmetry breaking after excitation. Upon relaxation, the energy of the broken-symmetry excited states significantly lowers, explaining the large observed Stokes shift. Moreover, in molecule 3, the novel phenomenon of symmetry swapping is observed.

**Fig. 5:**
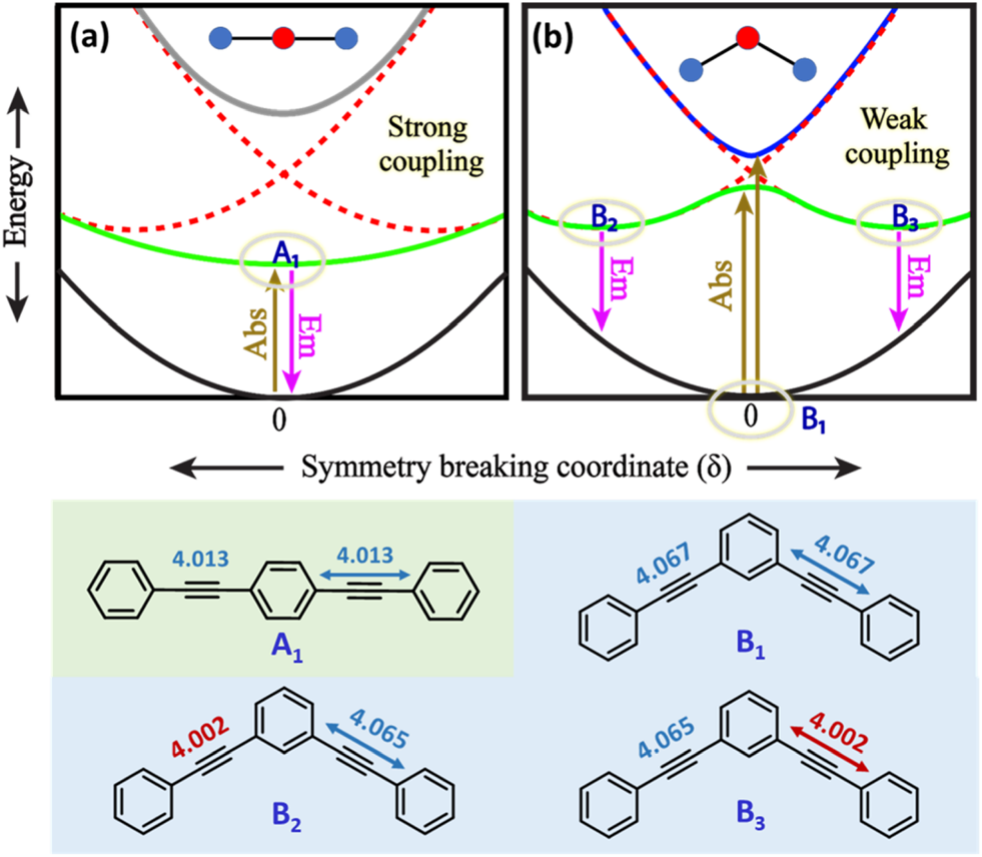
Qualitative sketch of potential energy surfaces for molecular systems as a function of an effective symmetry breaking coordinate, *δ*. Black and grey traces in panels (a) and (b) represent the ground state and dark excited states, respectively. The blue and green traces represent bright excited states of which the green trace is the emissive state. Panel (a) presents 2L in which symmetry is preserved. Due to strong coupling, the adiabatic potential energy surfaces of 2L have a single minimum and hence no symmetry breaking. Panel (b) presents 2B wherein symmetry breaking is seen. Due to weak coupling, the potential energy surface of the first excited state of 2B (green) has a double minimum and shows symmetry breaking. Bond lengths are reported in Å.

A detailed theoretical investigation shows that the three molecular systems undergoing excited-state symmetry breaking are characterized by the presence of degenerate (3 and 6) or quasi-degenerate (2B) excited states, a key feature for multistability. This point is illustrated in Fig. 5, showing a sketch of the potential energy surfaces relevant to 2L and 2B. The potential energy surfaces are plotted as a function of an effective symmetry-breaking coordinate, *δ*, that measures the asymmetry between the two arms. For 2L, all the potential energy surfaces are well-behaved, showing a single minimum at *δ* = 0: in all states the molecular symmetry is preserved. Instead for 2B, the potential energy surface relevant to the first excited-state shows a double minimum, so that fluorescence occurs from a distorted state with a finite *δ* value. Both systems have two equivalent arms, and the excited states can be associated with two diabatic states with the excitation localized on either arm. The potential energy surfaces relevant to the two diabatic states (dashed red lines in Fig. 5a and b) are two equivalent parabolas centered at equal and opposite *δ* values. In 2L, the strong interaction between the two arms, mediated by *para*-conjugation in the central benzene ring, is large enough to obliterate in the excited-state potential energy surface any memory of the two minima in the diabatic potential energy surface. In 2B, instead, the much weaker *meta*-conjugation leads to a potential energy surface with two equivalent minima for the first excited state. Therefore, upon light absorption, 2B is driven into a multistable excited-state: after excitation to a symmetric vertical state, the system rapidly relaxes towards one of the two equivalent minima, and the emission originating from the broken-symmetry state is largely red-shifted compared to absorption, explaining the large Stokes shift.

**Fig. 6:**
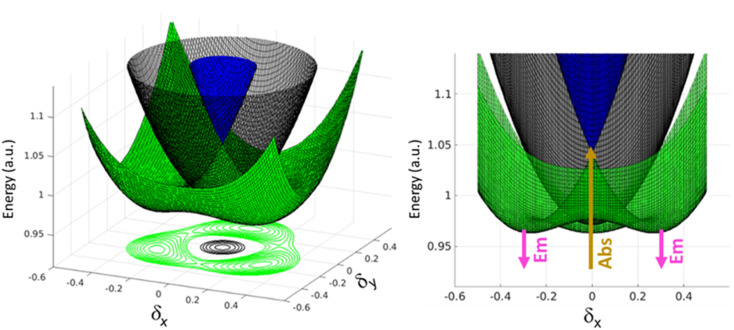
Qualitative sketch of potential energy surfaces of the first three excited states of molecule 3, plotted as a function of the two effective symmetry breaking coordinates *δ*_*x*_ and *δ*_*y*_. The left panel displays 3-dimensional potential energy surfaces and the front view is given in the right panel. The blue and green surfaces represent bright excited states of which the green trace is the emissive state. The grey surface is the dark state. The arrows in the right panels show the involved excited states in absorption and emission processes.

A similar but more complex picture applies to 3, where, as for 2B, the interaction between the three arms is weak due to *meta*-conjugation. The potential energy surfaces relevant to the three lowest electronic excited states of 3 are sketched in Fig. 6. For this three branched system, two symmetry-breaking coordinates must be introduced:^26,27^ indeed the three coordinates describing the molecular relaxation along each one of the three equivalent arms, *δ*_1_, *δ*_2_, and *δ*_3_, can be combined into a symmetric coordinate *δ*_+_ = *δ*_1_ + *δ*_2_ + *δ*_3_ that does not break the molecular symmetry, and two degenerate and mutually orthogonal symmetry-breaking coordinates *δ*_*y*_ = 2*δ*_1_ − *δ*_2_ − *δ*_3_ and δ_*x*_ = *δ*_2_ − *δ*_3_.

At *δ*_*x*_ = *δ*_*y*_ = 0, corresponding to the ground state geometry as relevant for absorption processes, the lowest excited-state is a dark state (grey in Fig. 6), while the two higher excited states (green and blue) are degenerate, as calculated in TDDFT. The green surface in Fig. 6 clearly shows multistability with three equivalent minima corresponding to broken-symmetry geometries. The symmetry swapping between a dark (grey) and a bright (green) state, promoted by symmetry breaking, determines both the strong emissivity of the system and the large Stokes shift.

## Conclusions

In conclusion, by combining experimental and computational methods, we have investigated the origin of the unusual photophysics exhibited by a series of multibranched PEs with different symmetries. The novel phenomenon of excited-state symmetry swapping is unveiled, explaining the observation of large fluorescence intensity in systems whose lowest-energy excitation is dark, without violating Kasha's rule. Among the molecules under investigation, *para*-conjugation promotes sizeable interactions among molecular branches in 2L and 4: large interactions lead to large splitting of the energy levels, *i.e.* to stable excited states well separated in energy. Multistability and symmetry breaking phenomena are observed in multibranched systems with degenerate or quasi-degenerate excited states. Degeneracy or quasi-degeneracy is observed in multibranched systems where the interaction among the different molecular arms is weak. Accordingly, symmetry breaking and/or symmetry swapping is observed in molecules 2B, 3 and 6, where *meta*-conjugation is responsible for weak interactions.

The large Stokes shifts observed for molecules 2B, 3 and 6 are a clear signature of excited-state symmetry breaking, with emission originating from a relaxed excited-state with non-equivalent arms. In addition to symmetry breaking, 3 exhibits the novel symmetry swapping phenomenon that shows up with the inversion of the energy order between a low-energy dark state and a high-energy bright state, thus providing an explanation to the strange photophysics of the system, without violating Kasha's rule. The lowest excited state has in fact a different nature at the ground state geometry (as relevant to absorption processes) and at the geometry of the first excited state (as relevant to fluorescence). Specifically, the lowest excited state is a dark state at the ground state geometry, suggesting a non-emissive behavior, but after excitation the molecule relaxes to a broken-symmetry geometry where the lowest excited state is bright, in a novel phenomenon dubbed here symmetry swapping. Molecule 6 is a more complex system, where multiple degenerate or quasi-degenerate excited states and low-energy multiple dark states are present. However, the concomitant presence of conjugation promotes the interaction between different arms: in this system, the lowering of symmetry upon excited-state relaxation is responsible for turning one of the dark states to bright.

The new phenomenon of excited-state swapping described here for a molecule belonging to the family of multibranched PEs can virtually occur in many systems having degenerate or quasi-degenerate excited states, prone to excited-state symmetry breaking. In multipolar dyes, symmetry-breaking is driven by polar solvation that largely stabilizes polar broken-symmetry states. Relevant dynamics, typically in the picosecond timescale, is easily addressed in pump–probe experiments. In contrast, in PEs, polar solvation plays a marginal role, and indeed symmetry swapping is also observed in a glassy solvent, where polar solvation is ineffective. Being not related to polar solvation, symmetry breaking and/or symmetry swapping in PE systems occurs in an ultrafast timescale (a few hundreds of fs) as related to vibrational relaxation, making time-resolved experiments more challenging.

The phenomenon of excited-state swapping provides an insight on some of the less understood photophysical properties in highly emissive multibranched conjugated systems with low energy dark excited states. The novel phenomenon of symmetry swapping presented herein can provide new directions in the design of multibranched molecular systems for optoelectronic applications.

## Experimental section

### UV-vis and fluorescence measurements at room temperature

Electronic absorption spectra were recorded using quartz cuvettes of 1 cm path length on a Shimadzu UV-3600 UV-vis-NIR spectrophotometer. Steady-state PL spectra were recorded on a Horiba Jobin Yvon fluorimeter, in a quartz cuvette of 1 cm path length.

### Low-temperature fluorescence

Low-temperature measurements were performed with an FLS1000 Edinburgh fluorometer, equipped with automatic polarizers, using a liquid nitrogen cooled optical cryostat (OptistatDN, Oxford Instruments) equipped with a temperature controller (ITC601, Oxford Instruments). 2-MeTHF (stored over molecular sieves for 1 night and filtered) and propylene glycol solutions were rapidly cooled down to 77 and 190 K, respectively, obtaining transparent glasses. Solutions for spectroscopic measurements were prepared using spectra grade or HPLC solvents. Fluorescence spectra have been corrected for the excitation intensity and the detector sensitivity.

### Two-photon absorption spectra

Two-photon absorption cross sections of 4 and 6 in chloroform were obtained by comparing their two-photon excited fluorescence (TPEF) intensity to that of a reference, a 6 × 10^−7^ M solution of fluorescein in water at pH > 10 (0.1 M NaOH), following a procedure described in the literature.^65^

The experimental setup consists of a Nikon A1R MP+ multiphoton upright microscope equipped with a Coherent Chameleon Discovery femtosecond pulsed laser (∼100 fs pulse duration with 80 MHz repetition rate, tunable excitation range 700–1300 nm). A 25× water dipping objective with a numerical aperture of 1.1 and 2 mm working distance was employed for focusing the excitation beam and for collecting the TPEF. The TPEF signal was directed by a dichroic mirror to a high sensitivity photomultiplier GaAsP detector, connected to the microscope through an optical fiber and preceded by a dispersive element. This detector allowed the spectral profile of the TPEF signal (wavelength range 400 to 620 nm for 4 and 6 or 430 to 650 nm for fluorescein, with a bandwidth of 10 nm) to be recorded. Correction for the wavelength dependent sensitivity of the detector was applied.

The measurements were carried out using 1 cm quartz cells placed horizontally under the microscope objective. Distilled water was employed to dip the objective and the focal point was moved as close as possible to the upper cuvette wall, at the same exact height for the reference and the samples. The concentrations of the sample solutions were 2 × 10^−5^ M and 8 × 10^−6^ M for 4 and 6, respectively. The corrected fluorescence spectra obtained by one- and two-photon excitation were well superimposed for all the investigated solutions, confirming that the emitting state is the same for both processes. Therefore, for each sample and for the reference, we assumed the same fluorescence quantum yield for one- and two-photon excited fluorescence.

Following the procedure reported in the literature, the two-photon absorption (TPA) cross section of the sample *σ*_2,new_ as a function of the excitation wavelength, *λ*, can be obtained as:^65^1
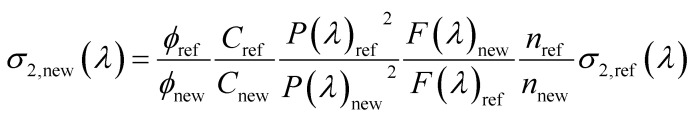


where *σ*_2,ref_ is the TPA cross section of the reference, *ϕ* is the fluorophore quantum yield, *C* is the solution concentration, *n* is the refractive index, *P*(*λ*) is the laser power at wavelength *λ*, and *F*(*λ*) is the integral of the TPEF spectrum, evaluated after correcting the emission spectrum for the detector sensitivity. The subscripts “new” and “ref” refer to the sample and to the reference, respectively. For each excitation wavelength, the quadraticity of the signal with respect to the excitation power was verified for all the solutions and the maximum deviation does not exceed 20%. The absolute values of *σ*_2,ref_(*λ*) of fluorescein were taken from the literature.^66^ TPA cross sections are expressed in Goeppert-Mayer units: 1 GM = 10^−50^ cm^4^ s photon^−1^.

### Transient absorption spectroscopy

The femtosecond transient absorption measurements of 2L and 6 in toluene were carried out by exciting the samples at 350 nm. The experimental setup comprises a Ti:sapphire amplified laser system: the output of a main oscillator (Mai Tai SP, Spectra Physics, 800 nm, 80 MHz) was used as a seeding for a regenerative amplifier, which generates amplified output pulses centered at 800 nm, with 5 mJ energy per pulse at 1 KHz repetition. From the amplified output, a pump pulse of 350 nm was generated using an optical parametric amplifier, and the residual pulse of 800 nm was passed through a motorized delay stage inside the pump–probe spectrometer. The white light continuum (WLC) was then generated by focusing this residual pulse into a rotating CaF_2_ plate of 2 mm thickness. A 1 : 1 beam splitter divides the WLC into probe and reference pulses. The pump power was maintained sufficiently low (2.5 mW) using neutral-density filters and the samples were continuously stirred in a rotating quartz cell with a 1.2 mm path length to avoid laser-induced photobleaching. A dual diode array detector with a 200 nm detection window was used to record the transient absorption spectra. The solvent responses were measured under the same experimental conditions (10% benzene in methanol) to determine the instrument response function (IRF), which was around 120 fs. The optical densities of the samples were kept at 0.6 at the excitation wavelength (*λ* = 350 nm) to improve the signal to noise ratio in the transient absorption spectra.

### DFT/TDDFT calculations

All TD-DFT calculations were performed using a Gaussian 16 computational^67^ package, using the CAM-B3LYP functional and 6-31G(d) basis set, in the gas phase. Initial molecular geometries were generated using GaussView 6.0 software.^55^ The geometry of all the PE derivatives was optimized before performing the vertical transition calculations. Optimized molecular structures and HOMO/LUMO electron density distributions were analyzed using GaussView 6.0 and Avogadro software.

Initial and final geometries in Fig. 3 and S7† correspond to the ground and excited state equilibrium geometries, respectively. Intermediate geometries are obtained moving the atoms along the displacement vector that connects the initial and final geometries.

## Author contributions

K. S., M. S., P. S. D.: synthesis and photophysical characterization; M. V.: transient absorption studies; A. D., F. T.: two-photon absorption and fluorescence anisotropy; A. L.: interpretation of photophysical studies; D. K. A. P. H, F. D. M., K. S., M. S.: computational studies. A. P., K. G. T., C. S.: conceptualization of the work; supervision; writing of the original draft. All authors contributed to review and editing of the draft.

## Conflicts of interest

There are no conflicts to declare.

## Supplementary Material

SC-014-D2SC05206G-s001
