# Supplementary material for: From symmetry breaking to symmetry swapping: is Kasha's rule violated in multibranched phenyleneethynylenes?
Source: Chem Sci. 2023 Jan 13;14(8):1986–96. doi: 10.1039/d2sc05206g (PMC9945429; doi:10.1039/d2sc05206g)
Supplement: SC-014-D2SC05206G-s001 [file SC-014-D2SC05206G-s001.pdf]

Electronic Supplementary Material (ESI) for Chemical Science.
This journal is © The Royal Society of Chemistry 2023

Supporting Information

From symmetry breaking to symmetry swapping: Is Kasha's rule violated in multibranched phenyleneethynylenes?

K. Swathi[‡ab], Meleppatt Sujith[‡b], P. S. Divya[b], Merin Varghese P,[b] Andrea Delledonne[a], D. K. Andrea Phan Huu[a], Francesco Di Maiolo[a], Francesca Terenziani[a], Andrea Lapini,[a] Anna Painelli[a], Cristina Sissa*[a], K. George Thomas*[b]

[a] Dipartimento di Scienze Chimiche, della Vita e della Sostenibilità Ambientale, Università di Parma, Parco Area delle Scienze 17A, 43124, Parma, Italy.

[b] School of Chemistry, Indian Institute of Science Education and Research Thiruvananthapuram (IISER TVM), Vithura, Thiruvananthapuram, 695 551, India.

Table of contents

1. **Synthesis and Characterization 3**

   1.1. Synthesis of Linear PE (2L) 4

   1.2. Synthesis of Bend PE (2B) 4

   1.3. Synthesis of Tripod PE (3) 5

   1.4. Synthesis of Tetrapod PE (4) 6

   1.5. Synthesis of Hexapod PE (6) 8

2. **Spectroscopic Measurements 10**

   2.1 Linear UV-Vis and Emission Spectra of DPA and 4 10

   2.2 Fluorescence Anisotropy Spectra: Revealing Multiple Excitations 12

   2.3 Transient absorption experiments of 2L and 6 14

3. **DFT/TDDFT Calculations 15**

   3.1 Orbital Analysis 21

   3.2 Optimized Excited State Geometry 24

   3.3 Optimized excited state geometries 25

4. **NMR Spectra of the compounds 29**

5. **References 38**

1. Synthesis and Characterization

All solvents and chemicals are purchased from commercial sources and used as received without further purification. All the precursors for the synthesis of the various phenyleneethynylene derivatives are purchased from Sigma Aldrich. **DPA** is purchased from Sigma Aldrich and used for various studies without further purification. Silica gel (200-400 mesh) is used to perform flash column chromatography. Final compounds are purified by passing through recycling HPLC manufactured by Japan Analytical Industry Co., Ltd. All melting points are uncorrected and determined using Stuart SMP30 melting point apparatus. ^{1}H and ^{13}C NMR spectra are recorded on Bruker Avans 500 MHz DPX spectrometer using 1,1,1,1-tetramethylsilane (TMS) as the internal standard. Elemental analysis is carried out on Elementar vario MICRO cube Elemental Analyser. Electronic absorption spectra are recorded using a quartz cuvette of 1 cm path length on a Shimadzu UV-3600 Vis-NIR Spectrophotometer. Steady-state PL spectra are recorded on Horiba Jobin Yvon Fluorimeter, in a quartz cuvette of 1 cm path length. Emission lifetimes are measured using a picosecond time-correlated single-photon counting system (model Horiba Jobin Yvon-IBH). Solutions are excited at 377 nm using a pulsed diode laser (NanoLED-375L; <100 ps pulse width). The detection system consists of a microchannel plate detector (model Hamamatsu R3809U-50) having an instrument response time of 38.6 ps, coupled to a monochromator (5000M) and TCSPC electronics (Data station Hub including Hub-NL, NanoLED controller).

1.1. Synthesis of Linear PE (2L)

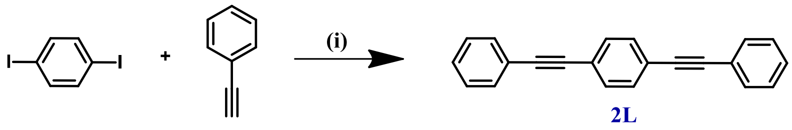

Scheme S1. *Synthesis of linear PE (**2L**); (i) $Pd(PPh_3)_2Cl_2$, CuI, TEA, THF.*

1,4-Diiodobenzene (500.00 mg, 1.515 mmol), $Pd(PPh_3)_2Cl_2$ (51.94 mg, 0.074 mmol) and CuI (8.57 mg, 0.045 mmol) are taken in a 2-neck round bottom flask and nitrogen is purged for 15 min. Triethylamine (5 mL), THF (5 mL) and phenylacetylene (371.35 mg, 3.636 mmol) are added and the reaction mixture is stirred at room temperature for 14 h. Solvent is evaporated under reduced pressure and the crude product is purified by silica gel column chromatography using petroleum ether as the eluent to obtain the pure product as a white solid (390 mg, 92.5%). mp 178-179 °C. ^1H NMR (500 MHz, $CDCl_3$, TMS) δ (ppm) 7.47–7.45 (m, 4H), 7.44 (s, 4H), 7.31-7.25 (m, 6H). ^{13}C NMR ($CDCl_3$, 125 MHz, TMS): δ (ppm) 131.65, 131.55, 128.48, 128.41, 123.12, 123.06, 91.24, 89.12. m/z (EI-MS) Calcd for $C_{22}H_{14}$, 278.11; Found, 278.15 $[M]^+$.

1.2. Synthesis of Bend PE (2B)

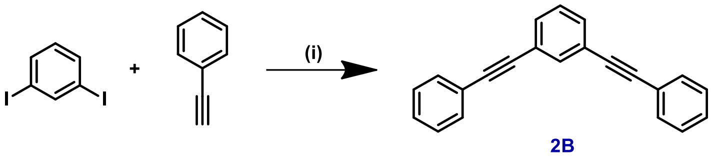

Scheme S2. *Synthesis of bent PE (**2B**). (i) $Pd(PPh_3)_2Cl_2$, CuI, TEA, THF.*

1,3-Diiodobenzene (500.00 mg, 1.515 mmol), $Pd(PPh_3)_2Cl_2$ (51.94 mg, 0.074 mmol) and CuI (42.85 mg, 0.225 mmol) are taken in a 2-neck round bottom flask and nitrogen is purged for 15 min. Triethylamine (5 mL), THF (5 mL) and phenylacetylene (371.35 mg, 3.636 mmol) are added and the reaction mixture is stirred at room temperature for 24 h. Solvent is evaporated under reduced

pressure and the crude product is purified by silica gel column chromatography using petroleum ether as the eluent to obtain the pure product (398 mg, 95%) as a white solid. mp 112-113 °C. ^{1}H NMR (500 MHz, $CDCl_3$, TMS) δ (ppm) 7.65 (s, 1H), 7.48-7.46 (dd, 4H), 7.43-7.41 (dd, 2H), 7.31-7.25 (m, 7H). ^{13}C NMR ($CDCl_3$, 125 MHz, TMS): δ (ppm) 133.58, 130.63, 130.25, 127.44, 127.42, 127.36, 122.61, 121.99, 88.94, 87.51. m/z (EI-MS) Calcd for $C_{22}H_{14}$, 278.11; Found, 278.15 $[M]^+$.

1.3. Synthesis of Tripod PE (3)

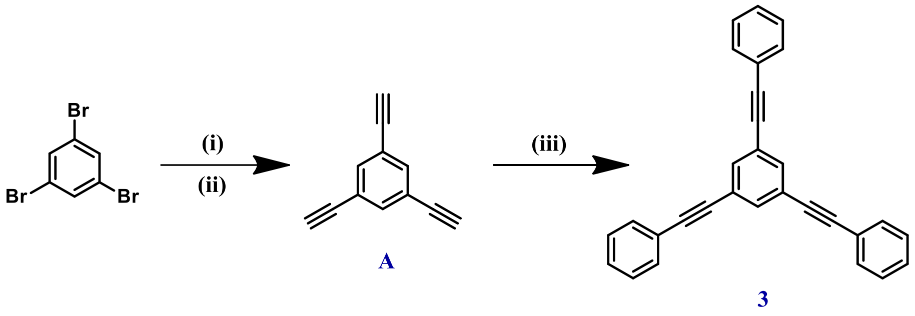

Scheme S3. *Synthesis of tripod PE (**3**). (i) TMSA, $Pd(PPh_3)_2Cl_2$, CuI, TEA ; (ii) NaOH, THF, MeOH; (iii) Iodobenzene, $Pd(PPh_3)_2Cl_2$, CuI, TEA, THF.*

Synthesis of 1,3,5-triethynylbenzene (A): 1,3,5-tribromobenzene (200.1 mg, 0.64 mmol), $Pd(PPh_3)Cl_2$ (13.0 mg, 0.02 mmol) and CuI (3.8 mg, 0.02 mmol) are taken in a pressure tube nitrogen is purged for 15 min. Triethylamine (7.0 mL), THF (7.0 mL), Trimethylsilylacetylene (TMSA) (0.36 mL, 2.55 mmol) are added and the reaction is stirred at 65 °C for 24 h. The solvent is evaporated under reduced pressure, extracted with dichloromethane, washed with water and brine, and dried over anhydrous sodium sulfate. The crude product obtained is purified by column chromatography using silica gel (hexane) to obtain 1,3,5-tris((trimethylsilyl)ethynyl)benzene as a pale-yellow solid (220.6 mg, 94%). Followed by this, 1,3,5-tris((trimethylsilyl)ethynyl)benzene (220 mg, 0.60 mmol) is dissolved in a mixture of THF (3.4 mL) and methanol (1.0 mL) and a solution of sodium hydroxide (0.13 mg in 1.0 mL of water) is added. The mixture is stirred at room temperature for 3 h. Solvent is evaporated, and extracted the residue with dichloromethane, washed with water and brine, and dried over anhydrous sodium sulfate to obtain the product as an off white solid (88.5 mg, 98%). mp

103-104 °C. ^{1}H NMR: δ (500 MHz, $CDCl_3$, TMS): 7.57 (s, 3H), 3.10 (s, 3H). ^{13}C NMR ($CDCl_3$, 125 MHz, TMS): δ (ppm) 135.67, 122.95, 81.63, 78.69. m/z (EI-MS) Calcd for $C_{12}H_6$, 150.05; Found, 150.10 $[M]^+$.

Synthesis of 1,3,5-tris(phenylethynyl)benzene (3): 1,3,5-triethynylbenzene (A) (50.00 mg, 0.324 mmol), $Pd(PPh_3)_2Cl_2$ (9.10 mg, 1.3 X 10^{-5} mmol) and CuI (3.08 mg, 1.621 X 10^{-5} mmol) are taken in a pressure tube and purged with nitrogen for 15 min. Iodobenzene (211.55 mg, 1.04 mmol), triethylamine (2 mL) and THF (8 mL) are added and the stirred the reaction mixture at 70° C for 14 h. Solvent is evaporated under reduced pressure and purified the crude product by column chromatography (silica gel) using a mixture (4:1) of pet ether and DCM to obtain the product as a white solid (123.76 mg, 57%). mp 147-148 °C. ^{1}H NMR (500 MHz, $CDCl_3$, TMS) δ (ppm) 7.59 (s, 3H), 7.48-7.46 (dd, 6H), 7.31-7.28 (m, 9H). ^{13}C NMR ($CDCl_3$, 125 MHz, TMS): δ (ppm) 133.02, 130.69, 127.59, 127.39, 123.02, 121.79, 89.49, 86.80. m/z (EI-MS) Calcd for $C_{30}H_{18}$, 378.14; Found, 378.15 $[M]^+$.

1.4. Synthesis of Tetrapod PE (4)

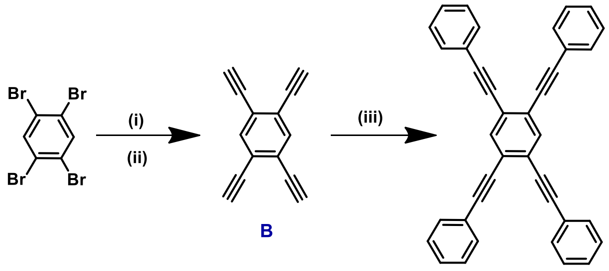

Scheme S4. *Synthesis of tetrapod PE (**4**). (i) TMSA, $Pd(PPh_3)_2Cl_2$, CuI, TEA ; (ii) NaOH, THF, MeOH; (iii) Iodobenzene, $Pd(PPh_3)_2Cl_2$, CuI, TEA, THF.*

***Synthesis of 1,2,4,5-tetraethynylbenzene (B)**:* 1,2,4,5-tetrabromobenzene (4.0 g, 10.3 mmol), $Pd(PPh_3)_2Cl_2$ (1.08 g, 1. 54 mmol) and CuI (0.29 g, 1. 54 mmol) are taken in a pressure tube and purged with nitrogen for 15 min. Triethylamine (50 mL) and TMSA (5.04 g, 51.3 mmol) are added, and stirred the reaction mixture at 70° C for 14 h. The solvent is evaporated under reduced pressure,

extracted with dichloromethane, washed with water and brine, and dried over anhydrous sodium sulfate. The crude product obtained is purified by column chromatography (silica gel) using a mixture (1:9) of DCM and petroleum ether to obtain 1,2,4,5-tetrakis((trimethylsilyl)ethynyl)benzene as a pale-yellow solid (4.5 g, 94.4%). mp 162-163 °C. ^{1}H NMR (500 MHz, $CDCl_3$, TMS) δ (ppm) 7.59 (s, 2H), 0.28 (s, 36H). ^{13}C NMR ($CDCl_3$, 125 MHz, TMS): δ (ppm) 136.11, 125.41, 102.09, 100.95. m/z (EI-MS) Calcd for $C_{26}H_{38}Si_4$, 462.21; Found, 462.15 $[M]^+$.

Followed by this, 1,2,4,5-tetrakis((trimethylsilyl)ethynyl)benzene (2.1 g, 4.3 mmol) is dissolved in a mixture of THF (12 mL) and MeOH (8 mL). A solution of NaOH (3.46 g in 18 mL water) is added and the reaction mixture was stirred at room temperature for 3 h. The solvent is evaporated under reduced pressure, extracted with dichloromethane, washed with water and brine, and dried over anhydrous sodium sulfate. The product obtained is purified by column chromatography (silica gel) using a mixture (1:1) of petroleum ether and DCM to obtain the product as a pale-brown solid (0.66 g, 89%). ^{1}H NMR (500 MHz, $CDCl_3$, TMS) δ (ppm) 7.66 (s, 2H), 3.45 (s, 4H). ^{13}C NMR ($CDCl_3$, 125 MHz, TMS): δ (ppm) 136.53, 125.25, 83.45, 80.46. m/z (EI-MS) Calcd for $C_{14}H_6$, 174.05; Found, 174.15 $[M]^+$.

Synthesis of 1,2,4,5-tetrakis(phenylethynyl)benzene (4): Compound B (50.00 mg, 0.287 mmol), $Pd(PPh_3)_2Cl_2$ (8.00 mg, 1.15 X 10^{-5} mmol) and CuI (2.70 mg, 1.417 X 10^{-5} mmol) are taken in a pressure tube and purged with nitrogen for 15 min. Iodobenzene (244.80 mg, 1.2 mmol), triethylamine (2 mL) and THF (8 mL) are added, and stirred the reaction mixture at 70° C for 14 h. The solvent is evaporated under reduced pressure, extracted with dichloromethane, washed with water and brine, and dried over anhydrous sodium sulfate. The crude product is purified by column chromatography (silica gel) using a mixture (1:9) of DCM and petroleum ether to obtain the product as a pale-yellow solid (90 mg, 65.5%). mp 189-190 °C. ^{1}H NMR (500 MHz, $CDCl_3$, TMS) δ (ppm) 7.70 (s, 2H), 7.52-7.50 (m, 8H), 7.31-7.29 (m, 12H). ^{13}C NMR ($CDCl_3$, 125 MHz, TMS): δ (ppm) 133.88,

130.72, 127.72, 127.43, 124.32, 121.95, 94.44, 86.52. m/z (EI-MS) Calcd for $C_{38}H_{22}$, 478.17; Found, 478.15 $[M]^+$.

1.5. Synthesis of Hexapod PE (6)

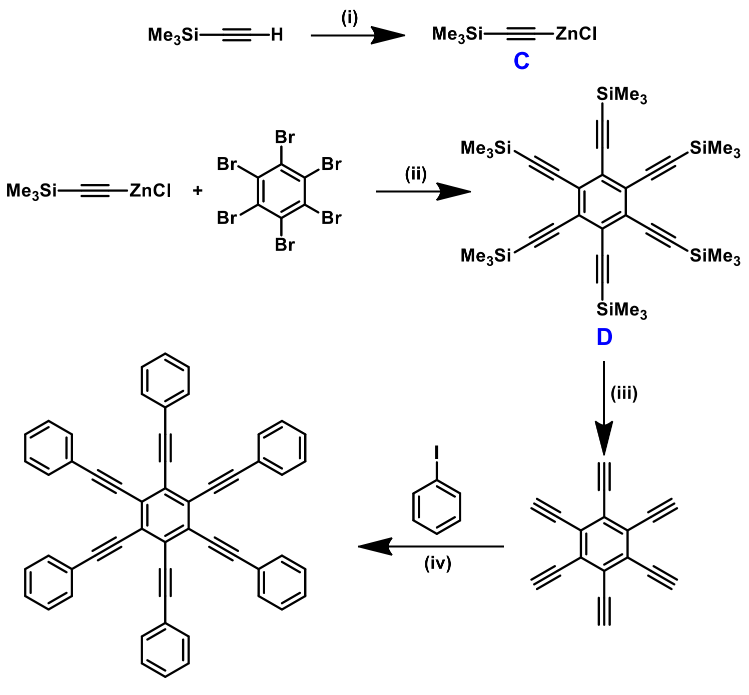

Scheme S5. *Synthesis of hexapod PE (**6**). (i) BuLi, THF, -78 °C, $ZnCl_2$, (ii) $Pd(PPh_3)_4$, THF, toluene, 80 °C, 72 h, (iii) K_2CO_3, THF, MeOH, (iv) $Pd(PPh_3)_4$, CuI, THF, toluene, TEA, rt, 24 h, 80 °C, 72 h.*

Synthesis of ((trimethylsilyl)ethynyl)zinc(III) chloride (C): To a solution of TMSA (20 mmol) in THF (10 mL) at -78 °C is added 20 mmol of n-butyllithium in hexane. The solution is stirred for 5 min followed by the addition of anhydrous zinc chloride (20 mmol) dissolved in THF (20 mL). The mixture is stirred for an additional 15 min at room temperature.

Synthesis of hexakis(trimethylsilylethynyl)benzene (D): To a three-necked flask, hexabromobenzene (552 mg 1.00 mmol), $Pd(PPh_3)_4$ (225 mg, 0.200 mmol), toluene (7 mL), and a solution of [(trimethylsilyl)ethynyl]zinc chloride (10.0 mmol) in THF(10 mL), prepared by the method mentioned above, are added in the sequence mentioned. The mixture is stirred under a nitrogen atmosphere at 80 °C for 72 h. After the addition of HCl (50 mL, 1 N), the reaction mixture

is extracted with ether (100 mL x 3). The combined organic layer is washed with brine (50 mL) and dried over $MgSO_4$. The solvent is evaporated, and the residue is purified by column chromatography (silica gel) using a mixture (1:12) of toluene and hexane to give hexa-trimethylsilylethynylbenzene (393.1 mg, 60%) as pale-yellow solid. ^1H NMR (500 MHz, $CDCl_3$, TMS) δ (ppm) 0.21 (s, 54H). ^{13}C NMR ($CDCl_3$, 125 MHz, TMS): δ (ppm) 127.95, 105.19, 100.99. m/z (EI-MS) Calcd for $C_{36}H_{54}Si_6$, 654.28; Found, 654.15 $[M]^+$.

Synthesis of 1,2,3,4,5,6-hexakis(phenylethynyl)benzene (6): To a degassed solution of hexakis(trimethylsilylethynyl)benzene (**6**) (118 mg, 0.180 mmol) in THF (10 mL) and methanol (10 mL) was added K_2CO_3 (248.0 mg,1.79 mmol) and water (0.1 mL). The resulting mixture is stirred at room temperature for 2 h. After the addition of water (30 mL), the mixture is extracted with diethyl ether, washed with water, and dried over Na_2SO_4. After removal of the solvents, dry THF (5.0 mL) and toluene (5.0 mL) are added immediately to the white solid while purging thoroughly with argon. To the solution, triethylamine (4.0 mL), iodobenzene (260 mg, 1.26 mmol) and CuI (6.0 mg, 63.0 µmol) is added. Finally, after the addition of $Pd(PPh_3)_4$ (62.0 mg, 0.107 mmol) to the degassed mixture, the mixture is stirred at room temperature for 24 h and 80 °C for an additional 24 h under argon atmosphere. The reaction mixture is diluted with dichloromethane, washed with brine, dried over Na_2SO_4, and concentrated. The crude mixture is purified by column chromatography (silica gel) using a mixture (1:4) of DCM and petroleum ether to obtain the product (51.3 mg, 52%) as a brownish solid. mp 169-170 °C. ^1H NMR (500 MHz, $CDCl_3$, TMS) δ (ppm) 7.59–7.57 (m, 12H), 7.32–7.29 (m, 18H). ^{13}C NMR ($CDCl_3$, 125 MHz, TMS): δ (ppm) 130.84, 127.91, 127.48, 126.52, 122.18, 98.40, 86.28. m/z (EI-MS) Calcd for $C_{54}H_{30}$, 678.23; Found, 678.50 $[M]^+$.

2. Spectroscopic Measurements

2.1 Linear UV-Vis and Emission Spectra of DPA and 4

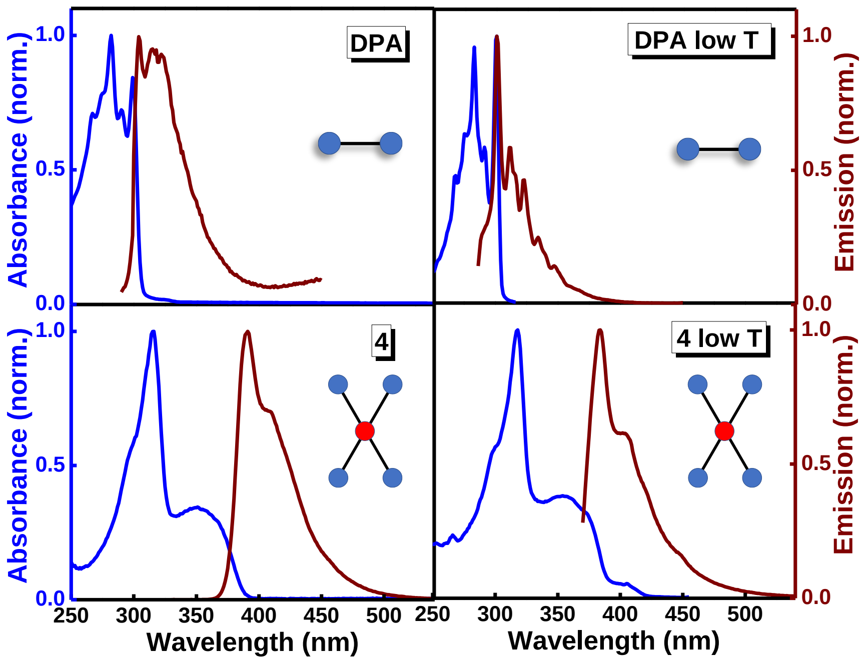

Figure S1: *Absorption/excitation (blue lines; at room temperature, absorption spectra were measured, while excitation spectra were measured at low temperature) and emission (brown lines) spectra of* ***DPA*** *and* ***4*** *molecules. Left column shows spectra measured in $CHCl_3$ solutions at room temperature while right column refers to results in propylene glycol at 190 K.*

Table S1: *Absorption and emission properties of* ***DPA*** *and* ***4*** *in $CHCl_3$*

Dye	λ_{abs} [a] [nm/cm^{-1}]	λ_{em} [b] [nm/cm^{-1}]	Stokes Shift[c] [cm^{-1}]	Molar extinction coefficient ($M^{-1}cm^{-1}$)	Quantum Yield	Lifetime (ns) [d]
**DPA**	299	304	550	2.76×10^4 *	<0.01	-
**4**	352	391	2834	3.44×10^4	0.75 ± 0.02	1.9

[a] first vibronic shoulder observed in the spectrum in $CHCl_3$; [b] first vibronic band of the fluorescence spectrum in $CHCl_3$; [c] the Stokes shift is estimated in $CHCl_3$ at room temperature; [d] excitation wavelength: 340nm
** From Ref [1] measured in 2-methyl pentane at 297 nm*

Table S2: *Experimental data in frozen solutions*

Dye[a]	λ_{exc} [nm][b]	λ_{em} [nm]	Stokes Shift [cm^{-1}]
**DPA**	299	299	0
**2L**	343	345	169
**2B**	302	327	2530
**3**	306	352	4270
**4**	371	384	912[c]
**6**	378	444	3932

[a]Propylene glycol at 190 K for all molecules except for 6 where 2-Me THF at 77 K is used due to solubility issues. [b]Maximum of the excitation spectrum. [c]The excitation spectrum of ***4*** *is broad at low temperature, hence the Stokes shift is estimated from the difference between the long-wavelength shoulder of absorption band peaked at 370 nm and the maximum of the emission band.*

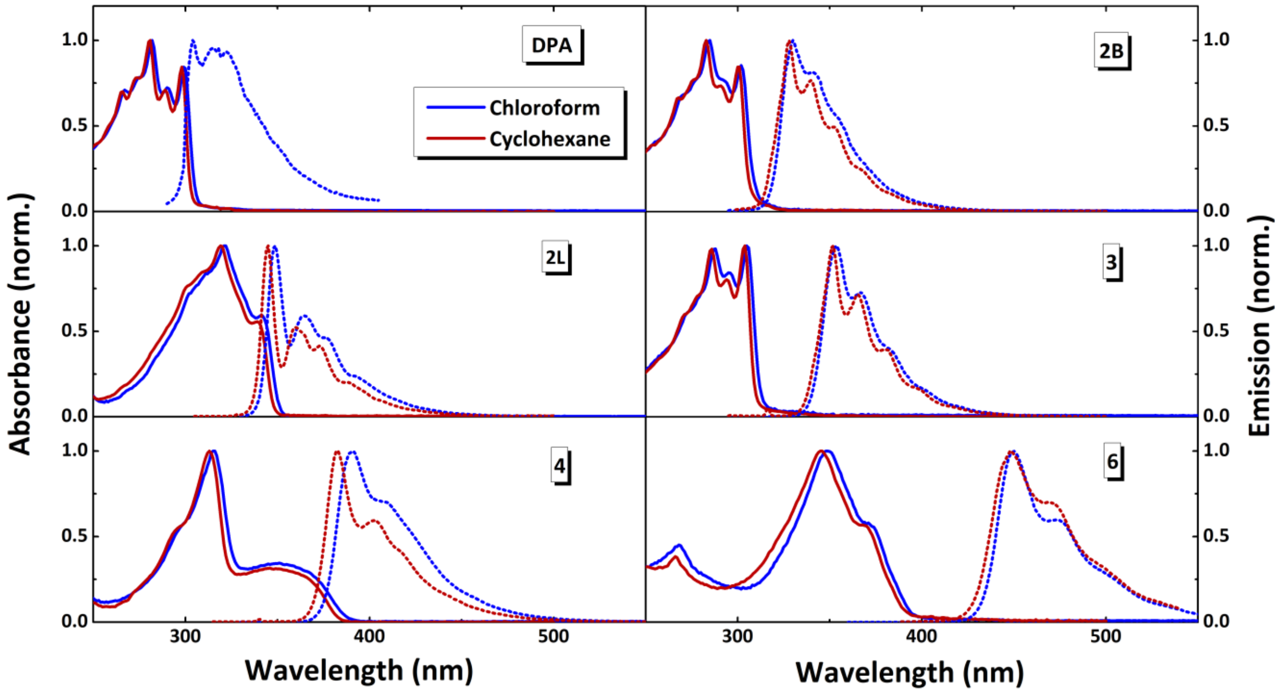

Figure S2: *Absorption (continuous lines) and emission (dotted lines) spectra of the family of PEs under investigation collected in chloroform and in cyclohexane.*

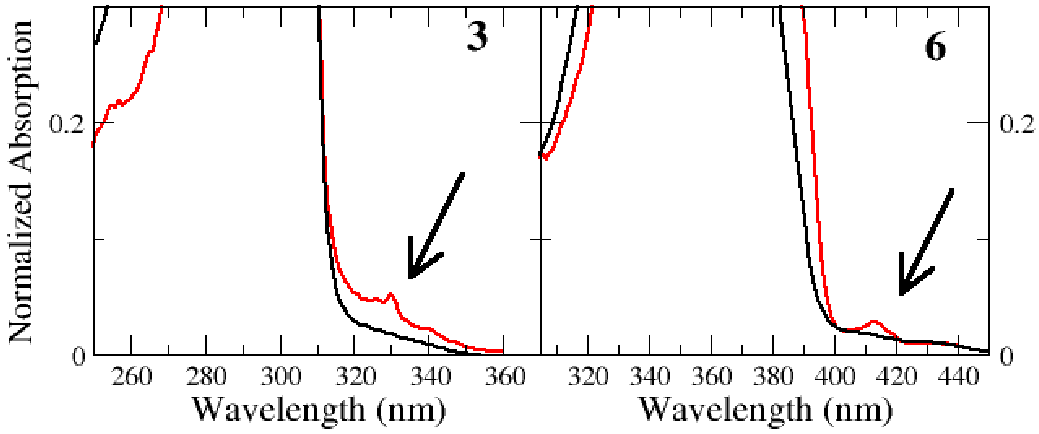

Figure S3: *Magnification of absorption spectra of **3** and **6** collected in $CHCl_3$ at room temperature (black lines) and excitations in frozen solutions at low temperature (red lines). In the spectral region between 320-360 nm for **3** and between 400-450 nm for **6** a very weak band is observed. The band is more resolved at low temperature due to the reduction of inhomogeneous broadening effects.*

2.2 Fluorescence Anisotropy Spectra: Revealing Multiple Excitations

Fluorescence anisotropy is measured by exciting the sample with vertically polarized light, and detecting emission through a polarizer oriented either parallel or perpendicular to the original polarization direction. Anisotropy is defined in Equation 1:

$$r = \frac{I_{\parallel} - I_{\perp}}{I_{\parallel} + 2I_{\perp}} \qquad (1)$$

where the denominator represents the total emission intensity. Fluorescence anisotropy provides information about the relative orientation of transition dipole moments responsible for absorption and emission. The fundamental anisotropy, r_0, is measured in the absence of depolarization induced by rotational diffusion (or by other sources of depolarization, such as energy transfer), and its value is related to the angle β between transition dipoles relevant to absorption and emission processes:

$$r_0 = \frac{2}{5}\left(\frac{3cos^2\beta - 1}{2}\right) \qquad (2)$$

Fundamental anisotropy varies from -0.2 (perpendicular dipoles) to 0.4 (collinear dipoles). Emission anisotropy is collected by exciting the sample at a fixed wavelength, and scanning emission wavelengths within the fluorescence band. Typically, the emission anisotropy is flat within the emission band that occurs from a single state, i.e., the lowest excited singlet. On the other hand, excitation anisotropy is collected by detecting emission intensity at a fixed wavelength while exciting the sample at varying wavelengths.

If multiple excited states contribute to absorption, and if their transition dipole moments are polarized along different directions, excitation anisotropy can acquire a wavelength dependence. Excitation anisotropy is exploited to resolve electronic spectra in the presence of multiple excitations which are very close in energy.[2] At any excitation wavelength λ the observed anisotropy can be expressed as:

$$r_0(\lambda) = \sum_{n=1}^{ex} f_n(\lambda) r_{0n} \qquad (3),$$

where the sum runs over the excited states, f_n is the fractional contribution of the *n*-th state to the total absorption at wavelength λ and r_{0n} represents the fundamental anisotropy of the *n*-th state. When the excitation involves degenerate states with the same oscillator strength, Equation 3 reduces to the average of the anisotropy of the excited states involved in the transition.

TDDFT results (Table S3) of **DPA** derivatives reveal the presence of multiple excited states lying very close in energy, and that are not resolved by UV-Vis absorption spectroscopy but can be resolved by fluorescence anisotropy in glassy solvents. Fig. S4 shows excitation anisotropy of **DPA** and **4** collected in glassy solvent (to avoid rotational diffusion), while excitation anisotropy spectra of **2L**, **2B**, **3** and **6** are reported in the main text.

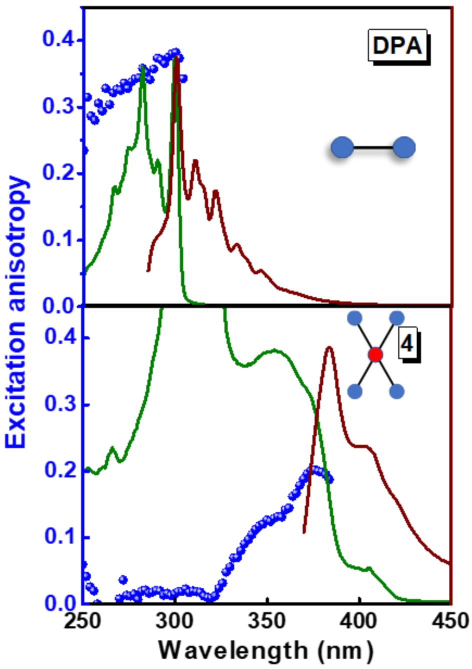

Figure S4. *Excitation (green lines), emission (brown lines) and excitation fluorescence anisotropy (blue spheres) of **DPA** and **4** (in propylene glycol at 190 K). Excitation wavelengths for anisotropy experiments: 320 nm (**DPA**); 460 nm (**4**)*

2.3 Transient absorption experiments of 2L and 6

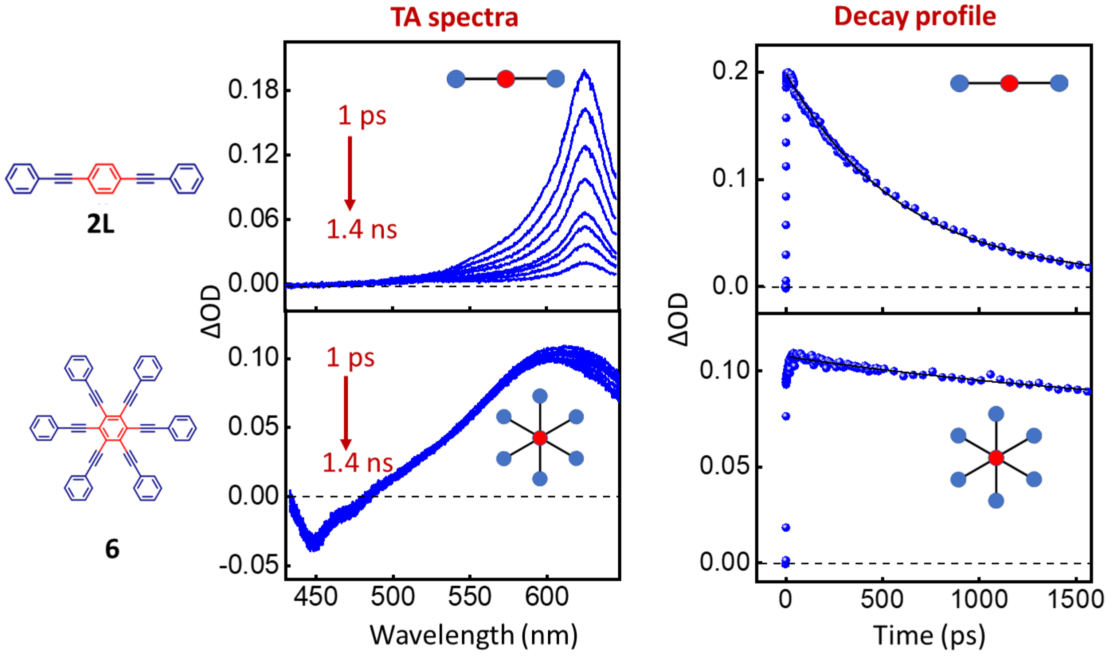

Figure S5. *Transient absorption spectra (left) in the spectral window of 420 nm – 650 nm of* ***2L*** *and* ***6*** *and the corresponding decay profiles (right) in the time window of 1.5 ns in toluene. Excitation wavelength: 350 nm.*

3. DFT/TDDFT Calculations

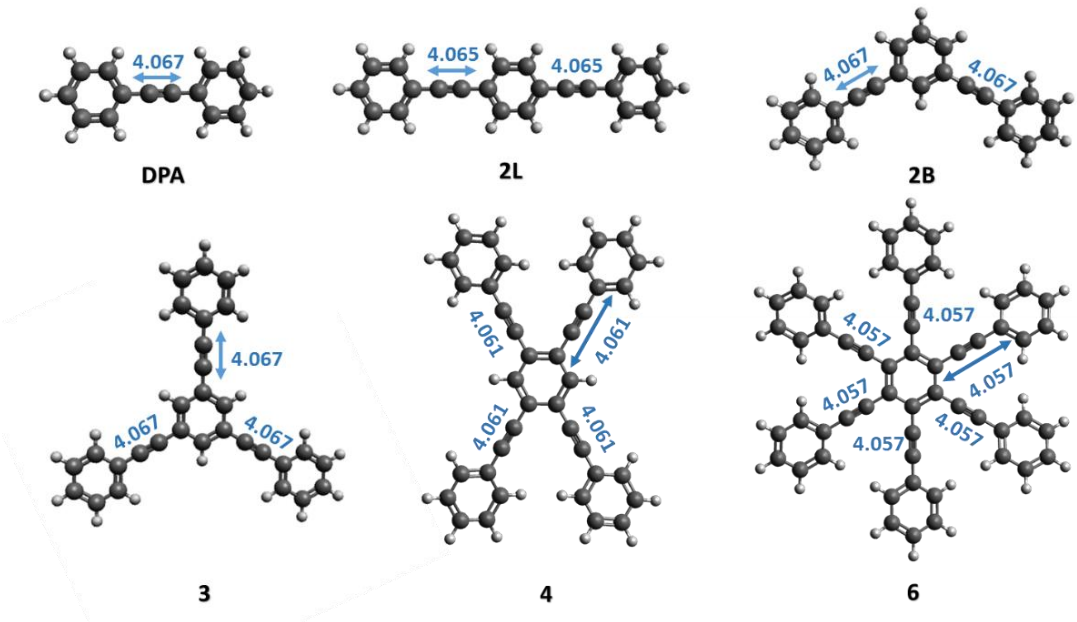

Figure S6*: Optimized ground-state structures. Bond lengths are reported in Å.*

Table S3: *TDDFT results at the optimized ground state geometry. The first 10 transitions are reported for all the molecules*

Compound	Transition number	Transition wavelength (nm)	Oscillator strength	Transition dipole moments (a.u.)	Orbitals & coefficients
**DPA**	1	271.64	0.90	x: -2.84<br>y: 0.00<br>z: 0.00	HOMO -> LUMO (0.68)
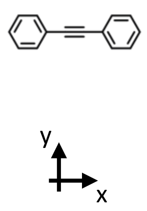	2	237.77	0.00	x: 0.00<br>y: 0.03<br>z: 0.00	HOMO -> LUMO+1 (0.45)<br>HOMO-1 -> LUMO (-0.44)
	3	237.54	0.00	x: 0.00<br>y: 0.00<br>z: 0.00	HOMO -> LUMO+2 (0.45)<br>HOMO-2 -> LUMO (-0.44)
	4	237.39	0.00	x: 0.00	(HOMO-4) -> LUMO (0.69)

				y: 0.00<br>z: 0.00	
	5	201.26	0.00	x: 0.00<br>y: 0.00<br>z: 0.00	HOMO -> LUMO+4 (0.67)
	6	200.11	0.00	x: 0.00<br>y: 0.00<br>z: 0.00	HOMO -> LUMO+3 (0.48)
	7	193.47	0.00	x: 0.00<br>y: 0.01<br>z: 0.00	HOMO -> LUMO+2 (0.51)<br>HOMO-2 -> LUMO (0.48)
	8	192.48	0.37	x: 0.00<br>y: -1.54<br>z: 0.00	HOMO-1 -> LUMO (0.48)<br>HOMO -> LUMO+1 (0.51)
	9	189.55	0.00	x: 0.00<br>y: 0.00<br>z: 0.00	HOMO-3 -> LUMO (0.51)<br>HOMO -> LUMO+3 (-0.46)
	10	182.65	0.29	x: 1.33<br>y: 0.00<br>z: 0.00	HOMO-2 -> LUMO+2 (0.43)<br>HOMO-1 -> LUMO+1 (0.43)
**2L**	1	313.38	1.91	x: -4.44<br>y: 0.00<br>z: 0.00	HOMO -> LUMO (0.67)
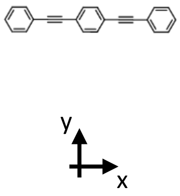	2	251.50	0.00	x: 0.00<br>y: -0.03<br>z: 0.00	HOMO -> LUMO+2 (0.48)<br>HOMO-4 -> LUMO (0.46)
	3	250.01	0.00	x: 0.00<br>y: 0.00<br>z: 0.00	HOMO-1 -> LUMO (0.55)
	4	243.24	0.00	x: 0.00<br>y: 0.00<br>z: 0.00	HOMO-5 -> LUMO (0.63)
	5	241.56	0.00	x: 0.00<br>y: 0.00<br>z: 0.00	HOMO-6 -> LUMO (0.63)
	6	239.03	0.00	x: 0.00<br>y: -0.04<br>z: 0.00	HOMO-2 -> LUMO (0.36)<br>HOMO -> LUMO+4 (0.35)
	7	239.02	0.00	x: 0.00<br>y: -0.04<br>z: 0.00	HOMO-3 -> LUMO (0.36)<br>HOMO -> LUMO+3 (-0.35)

	8	219.74	0.00	x: 0.00<br>y: 0.00<br>z: 0.00	HOMO -> LUMO+1 (0.55)
	9	204.44	0.00	x: 0.00<br>y: 0.00<br>z: 0.00	HOMO -> LUMO+6 (0.60)
	10	203.83	0.03	x: 0.46<br>y: 0.00<br>z: 0.00	HOMO -> LUMO+5 (0.39)<br>HOMO-1 -> LUMO+1 (-0.38)
**2B**	1	275.33	1.66	x: 3.88<br>y: 0.01<br>z: 0.00	HOMO-1 -> LUMO (0.52)<br>HOMO -> LUMO+1 (0.45)
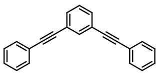 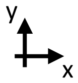	2	271.16	0.36	x: 0.01<br>y: -1.79<br>z: 0.00	HOMO -> LUMO (0.61)
	3	263.75	0.01	x: 0.24<br>y: 0.00<br>z: 0.00	HOMO -> LUMO+1 (0.44)<br>HOMO-1 -> LUMO (-0.37)
	4	237.98	0.00	x: 0.00<br>y: 0.00<br>z: 0.00	HOMO-5 -> LUMO (0.56)<br>HOMO-7 -> LUMO+1 (-0.40)
	5	237.49	0.00	x: -0.02<br>y: 0.03<br>z: 0.00	HOMO-2 -> LUMO (0.28)<br>HOMO-2 -> LUMO+1 (-0.28)<br>HOMO-1 -> LUMO+2 (-0.28)<br>HOMO -> LUMO+2 (0.28)
	6	237.48	0.00	x: 0.02<br>y: 0.03<br>z: 0.00	HOMO-3 -> LUMO (0.28)<br>HOMO-3 -> LUMO+1 (0.28)<br>HOMO-1 -> LUMO+3 (-0.28)<br>HOMO -> LUMO+3 (-0.28)
	7	236.89	0.00	x: 0.00<br>y: 0.00<br>z: 0.00	HOMO-7 -> LUMO (0.51)<br>HOMO-5 -> LUMO+1 (-0.45)
	8	222.02	0.01	x: 0.00<br>y: 0.32<br>z: 0.00	HOMO-1 -> LUMO+1 (0.61)
	9	201.91	0.00	x: 0.00<br>y: 0.01<br>z: 0.00	HOMO -> LUMO+6 (0.54)<br>HOMO-1 -> LUMO+7 (-0.40)
	10	201.81	0.03	x: 0.00<br>y: 0.41<br>z: 0.00	HOMO-1 -> LUMO+4 (0.38)<br>HOMO -> LUMO+5 (0.33)

**3**	1	285.81	0.00	x: 0.00<br>y: 0.00<br>z: 0.00	HOMO-1 -> LUMO+1 (0.42)<br>HOMO -> LUMO (0.42)
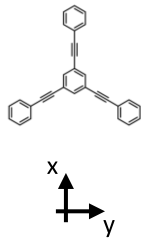	2	277.27	1.61	x: -1.99<br>y: -3.27<br>z: 0.00	HOMO-1 -> LUMO (0.37)<br>HOMO -> LUMO+1 (0.37)
	3	277.27	1.61	x: 3.27<br>y: -1.99<br>z: 0.00	HOMO-1 -> LUMO+1 (0.37)<br>HOMO -> LUMO (-0.37)
	4	270.05	0.00	x: 0.01<br>y: 0.00<br>z: 0.00	HOMO-1 -> LUMO (0.43)<br>HOMO -> LUMO+1 (-0.43)
	5	238.37	0.00	x: 0.00<br>y: 0.00<br>z: 0.00	HOMO-6 -> LUMO (0.48)
	6	238.37	0.00	x: 0.00<br>y: 0.00<br>z: 0.00	HOMO-6 -> LUMO+1 (0.48)
	7	237.40	0.00	x: 0.02<br>y: -0.07<br>z: 0.00	HOMO-2 -> LUMO+4 (-0.25)<br>HOMO-3 -> LUMO (0.27)
	8	237.40	0.00	x: 0.07<br>y: -0.02<br>z: 0.00	HOMO-2 -> LUMO+3 (0.26)<br>HOMO-5 ->LUMO+1 (-0.24)
	9	229.13	0.00	x: 0.03<br>y: -0.01<br>z: 0.00	HOMO-2 -> LUMO (0.51)
	10	229.13	0.00	x: -0.01<br>y: -0.03<br>z: 0.00	HOMO-2 -> LUMO+1 (0.51)
**4**	1	347.81	0.69	x: 0.00<br>y: 2.81<br>z: 0.00	HOMO -> LUMO (0.67)
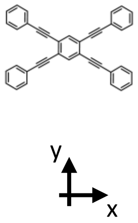	2	310.80	0.47	x: -2.20<br>y: 0.00<br>z: 0.00	HOMO-1 -> LUMO (0.61)<br>HOMO -> LUMO+1 (0.25)
	3	293.49	2.48	x: 4.89<br>y: 0.00<br>z: 0.00	HOMO-1 -> LUMO (-0.27)<br>HOMO -> LUMO+1 (0.61)
	4	286.32	0.00	x: 0.00<br>y: 0.00	HOMO-2 -> LUMO (0.58)<br>HOMO -> LUMO+2 (0.31)

				z: -0.13	
	5	256.10	0.00	x: 0.00<br>y: 0.00<br>z: -0.03	HOMO-9 -> LUMO (0.57)
	6	255.06	0.00	x: 0.00<br>y: -0.10<br>z: 0.00	HOMO-8 -> LUMO (0.55)
	7	249.51	0.00	x: 0.00<br>y: 0.00<br>z: 0.00	HOMO-11 -> LUMO (-0.35)<br>HOMO-3 -> LUMO (0.44)
	8	249.10	0.02	x: -0.38<br>y: 0.00<br>z: 0.00	HOMO-12 -> LUMO (-0.31)<br>HOMO-10 -> LUMO (0.49)
	9	241.63	0.00	x: 0.00<br>y: -0.08<br>z: 0.00	HOMO-1 -> LUMO+1 (0.66)
	10	240.14	0.00	x: 0.00<br>y: 0.00<br>z: 0.04	HOMO -> LUMO+5 (+0.28)<br>HOMO-5 -> LUMO (-0.28)
**6**	1	362.97	0.00	x: 0.00<br>y: 0.00<br>z: 0.00	HOMO-1 -> LUMO (0.47)<br>HOMO -> LUMO+1 (0.47)
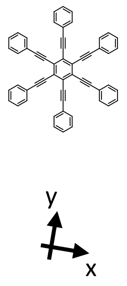	2	361.43	0.00	x: 0.00<br>y: 0.00<br>z: 0.00	HOMO-1 -> LUMO+1 (-0.47)<br>HOMO -> LUMO (0.47)
	3	328.88	1.88	x: 1.10<br>y: -4.38<br>z: 0.00	HOMO-1 -> LUMO (0.26)<br>HOMO-1 -> LUMO+1 (0.40)<br>HOMO -> LUMO (0.40)<br>HOMO -> LUMO+1 (-0.26)
	4	328.87	1.88	x: 4.38<br>y: 1.10<br>z: 0.00	HOMO-1 -> LUMO (0.40)<br>HOMO-1 -> LUMO+1 (-0.26)<br>HOMO -> LUMO (-0.26)<br>HOMO -> LUMO+1 (-0.40)
	5	297.18	0.00	x: 0.00<br>y: 0.00<br>z: 0.00	HOMO-2 -> LUMO (0.54)<br>HOMO -> LUMO+2 (0.25)
	6	297.18	0.00	x: 0.00<br>y: 0.00<br>z: 0.00	HOMO-2 -> LUMO+1 (0.54)<br>HOMO-1 -> LUMO+2 (-0.25)
	7	268.37	0.00	x: 0.00<br>y: 0.00	HOMO-6 -> LUMO (0.55)

				z: 0.00	
	8	268.37	0.00	x: 0.00<br>y: 0.00<br>z: 0.00	HOMO-6 -> LUMO+1 (0.55)
	9	248.59	0.00	x: 0.00<br>y: 0.00<br>z: 0.00	HOMO-2 -> LUMO (-0.30)<br>HOMO -> LUMO+2 (0.39)
	10	248.59	0.00	x: 0.00<br>y: 0.00<br>z: 0.00	HOMO-2 -> LUMO+1 (0.30)<br>HOMO-1 -> LUMO+2 (0.39)

Table S4*: Orbital energies*

Compound	Orbital	Energy (eV)
**DPA**	HOMO	-7.025
	LUMO	-0.003
**2L**	HOMO	-6.717
	LUMO	-0.547
**2B**	LUMO+1	0.124
	LUMO	-0.232
	HOMO	-6.982
	HOMO-1	-7.292
**3**	LUMO+2	0.417
	LUMO+1	-0.291
	LUMO	-0.291
	HOMO	-7.058
	HOMO-1	-7.058
	HOMO-2	-7.573
**4**	LUMO+1	-0.116
	LUMO	-0.938
	HOMO	-6.567
	HOMO-1	-7.178
**6**	LUMO+2	0.169
	LUMO+1	-0.936
	LUMO	-0.936
	HOMO	-6.583
	HOMO-1	-6.583
	HOMO-2	-7.329

3.1 Orbital Analysis

DPA and **2L** have a single bright transition at low energy, polarized along the main molecular axis, with a dominant HOMO to LUMO character.

As for **2B**, four orbitals (HOMO-1, HOMO, LUMO and LUMO+1) are mainly involved in the first two transitions (Table S3). The poor conjugation in the bent system leads to quasi-degenerate HOMO and HOMO-1 orbitals as well as LUMO and LUMO+1 (Table S4). As a result, the two low-energy transitions of **2B** are very close in energy (Table S3). Similarly, the lowest energy transitions in **4** involve the HOMO-1, HOMO, LUMO and LUMO+1 orbitals. However, in contrast to **2B**, HOMO/HOMO-1 and LUMO/LUMO+1 orbitals in **4** are well separated in energy (Table S4).

More interestingly, the detailed analysis of the orbitals involved in the low-energy transitions of **3** and **6**, provide an explanation of the presence of dark states. Starting with **3**, we can roughly describe it as a multibranched system composed of three branches, each branch corresponding to **DPA** molecule. Since we are only interested in low energy transitions, the HOMO and LUMO (denoted as H and L, respectively) orbitals of **DPA** are of significance in this context. Considering molecule **3**, the three HOMO orbitals of each branch, H_1, H_2, H_3, recombine to give a totally symmetric orbital, (A_1' representation using the notation for D_{3h}) and a pair of degenerate orbitals (E' representation). Similarly, the three local LUMO orbitals, L_1, L_2, L_3 recombine into two orbitals of E' symmetry and one orbital of A_1' (Scheme S1).

a)

D_{3h}	E	$2C_3$	$3C_2'$	σ_h	$2S_3$	$3\sigma_v$
Γ	3	0	1	3	0	1

b)

$$\Gamma_{A_1'} = \frac{1}{12}(3+3+3+3) = 1$$

$$\Gamma_{A_2'} = \frac{1}{12}(3-3+3-3) = 0$$

$$\Gamma_{E'} = \frac{1}{12}(6+6) = 1$$

$$\Gamma_{A_1''} = \frac{1}{12}(3+3-3-3) = 0$$

$$\Gamma_{A_2''} = \frac{1}{12}(3-3-3+3) = 0$$

$$\Gamma_{E''} = \frac{1}{12}(6-6) = 0$$

$$\boldsymbol{\Gamma = A_1' \oplus E'}$$

c)

$$\phi_{A_1'} = \frac{1}{\sqrt{3}}(H_1 + H_2 + H_3)$$

$$\phi^1_{E'} = \frac{1}{\sqrt{6}}(2H_1 - H_2 - H_3)$$

$$\phi^2_{E'} = \frac{1}{\sqrt{2}}(H_2 - H_3)$$

Scheme S6: *Symmetrization of frontier orbitals for **3**. Panel a): Characters of the reducible representation Γ. The basis is constituted by the three HOMO orbitals of **DPA**, one for of each branch, H_1, H_2, H_3. Panel b): Reduction of the representation Γ . Panel c) Symmetry adapted linear combinations (SALCs) of basis states. The same procedure applies to LUMO orbitals*

Table S5 shows the symmetrized linear combinations of orbitals of **DPA**, and the comparison with the shape of orbitals of **3**: the proposed picture that maps the orbitals of **3** as linear combinations of orbitals of **DPA** is fully consistent with TDDFT results: HOMO-1/HOMO (LUMO/LUMO+1) is the pair of degenerate E'-symmetry orbitals of **3**, while HOMO-2 (LUMO+2), lying close to HOMO (and LUMO) have A_1' symmetry.

Table S5: *Orbital analysis of **3***

**HOMO of DPA (H)**	**LUMO of DPA (L)**
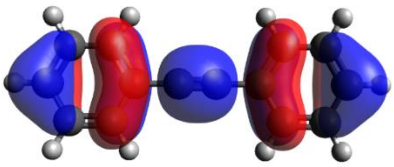	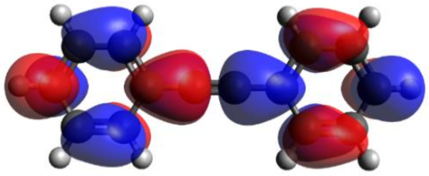

**Linear combinations of HOMOs orbitals of DPA**	**Orbital shapes of 3**	**Linear combinations of LUMOs orbitals of DPA**	**Orbital shapes of 3**
$\frac{1}{\sqrt{3}}(H_1 + H_2 + H_3)$ A'$_1$-symmetry	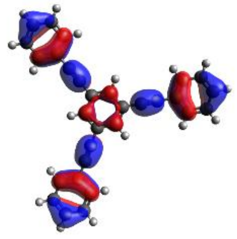 HOMO-2	$\frac{1}{\sqrt{3}}(L_1 + L_2 + L_3)$ A'$_1$-symmetry	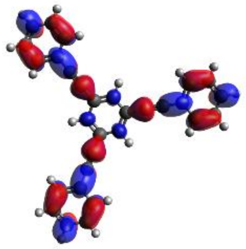 LUMO+2
$\frac{1}{\sqrt{2}}(H_2 - H_3)$ E'-symmetry	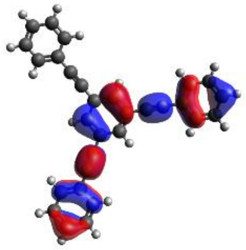 HOMO-1	$\frac{1}{\sqrt{2}}(L_2 - L_1)$ E'-symmetry	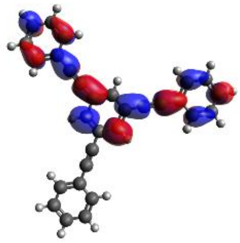 LUMO +1
$\frac{1}{\sqrt{6}}(2H_1 - H_2 - H_3)$ E'-symmetry	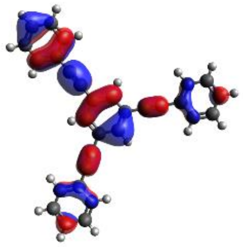 HOMO	$\frac{1}{\sqrt{6}}(2L_3 - L_1 - L_2)$ E'-symmetry	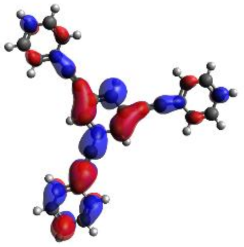 LUMO

The lowest transitions in **3** arise from the two pairs of degenerate HOMO and LUMO orbitals that will give rise to four transitions whose symmetry is obtained from the direct product of the two degenerate representations:

$$E' \otimes E' = E' + A_1{}' + A_2{}'$$

Both the two non-degenerate transitions (A_1' and A_2') are forbidden by symmetry. These two states correspond to the dark state labeled as 1 and 4 in Table S3. The two-fold degenerate state (E'-symmetry, state 2 and 3 in Table S3) are bright and polarized along perpendicular directions. Of course, sizable contributions to these E'-transitions are also given by other excitations of E'-

symmetry arising from the same group of orbitals, specifically the HOMO-2 to LUMO or LUMO+1 excitations and HOMO-1 or HOMO to LUMO+2.

A similar analysis, even if more complex, can be done on molecule **6**, where the presence of six equivalent arms again leads to pairs of degenerate orbitals. To keep things simple, we make reference to the D_{3h} subgroup, and we expect that out of the six equivalent H orbitals in each molecular branch, one obtains two non-degenerate orbitals (A_1' symmetry) and two pairs of degenerate orbitals (E' symmetry). Exactly the same result is obtained from the six equivalent L orbitals. The major difference with respect to molecule **3** is that para-conjugation in **6** leads to larger splitting of the non-degenerate orbitals (Table S4). In any case, for **6**, two dark states are expected lying below the allowed degenerate excited states. The proposed analysis of orbitals explains the origin of dark states as well as of degenerate states in molecules that, like **3** and **6**, have a high symmetry axis (C_3 and C_6, respectively).

3.2 Optimized Excited State Geometry

Table S6. *Selected TDDFT results obtained from the optimized excited state geometry of each compound*

Compound	Optimized excited state	Transition wavelength	Oscillator Strength	Nature of the transition
**DPA**	1	318	0.93	HOMO -> LUMO (0.68)
**2L**	1	363	1.99	HOMO -> LUMO (0.68)
	2	266	0.00	HOMO-2 -> LUMO (0.46)<br>HOMO -> LUMO+2 (-0.49)
**2B**	1	319	1.13	HOMO -> LUMO (-0.64)
	2	319	1.13	HOMO -> LUMO (0.64)
**3**	1	305	0.00	HOMO-1 -> LUMO+1 (-0.46)<br>HOMO -> LUMO (0.46)
	2	321	1.24	HOMO -> LUMO (0.64)
	3	321	1.24	HOMO -> LUMO (0.64)

	4	282	0.00	HOMO-1 -> LUMO+1 (0.46)<br>HOMO -> LUMO (0.46)
**4**	1	396	0.65	HOMO -> LUMO (0.68)
	2	334	0.50	HOMO-1 -> LUMO (0.62)<br>HOMO -> LUMO+1 (0.24)
	3	314	2.24	HOMO-1 -> LUMO (0.28)<br>HOMO -> LUMO+1 (0.58)
	4	307	0.01	HOMO-2 -> LUMO (0.59)<br>HOMO -> LUMO+2 (0.32)
**6**	1	391	0.00	HOMO-1 -> LUMO (-0.48)<br>HOMO -> LUMO+1 (0.48)
	2	394	0.36	HOMO-2 -> LUMO+2 (0.12)<br>HOMO-1 -> LUMO+1 (0.24)<br>HOMO -> LUMO (-0.64)
	3	349	1.85	HOMO-1 -> LUMO (-0.49)<br>HOMO -> LUMO+1 (0.47)
	4	349	1.85	HOMO-1 -> LUMO (0.49)<br>HOMO -> LUMO+1 (0.47)

3.3 Optimized excited state geometries

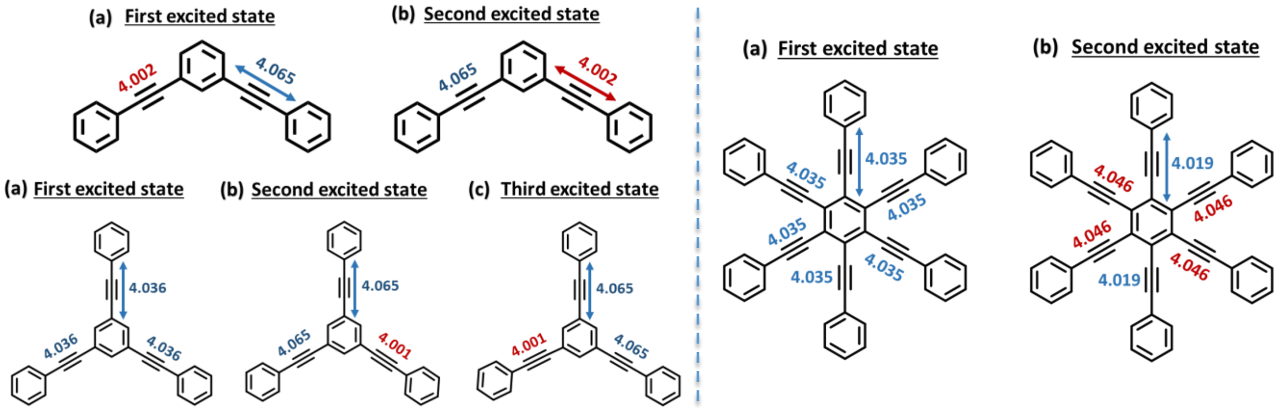

Figure S7. *Optimized excited state geometry of molecules* ***2B****,* ***3*** *and* ***6****; for each dye, geometries (a), (b) and (c) are obtained upon optimization of the first, second and third vertical excited state, respectively. Bond lengths are reported in Å.*

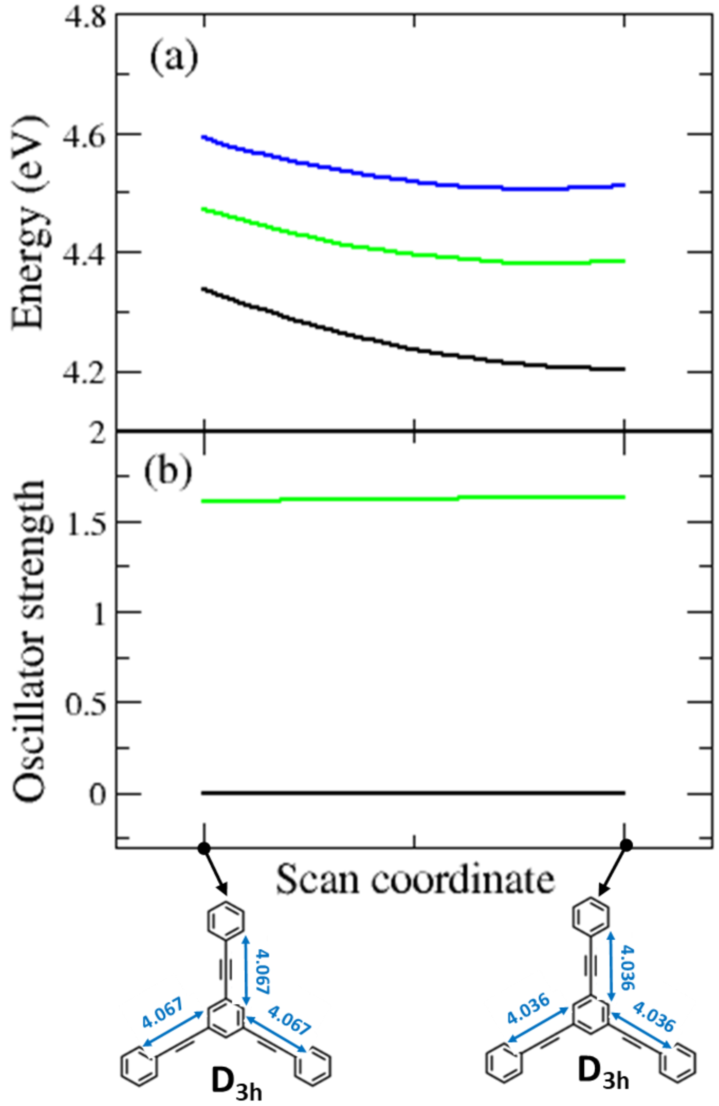

***Figure S8**. Results of scan calculations performed on molecules **3**, where symmetry swapping occurs. The start and end points of the calculation are reported on the x-axis, corresponding to the optimized ground state geometry and the optimized excited state geometry, respectively. The scan is performed by defining an effective coordinate connecting the ground state geometry, x_{gs}, to the excited state geometry, x_{es}. Intermediate geometries are obtained moving the atoms from x_{gs} along the displacement vector $d = x_{es}$-x_{gs}. The symmetry group is D_{3h} for both structures, since symmetry is preserved in this case. Panel a shows the evolution of the energy of the four lowest excited states (actually the green line corresponds to two degenerate states). Panel b shows corresponding oscillator strengths (again the green line refers to the two degenerate states), while both the blue and the black states are dark. Bond lengths are reported in Å.*

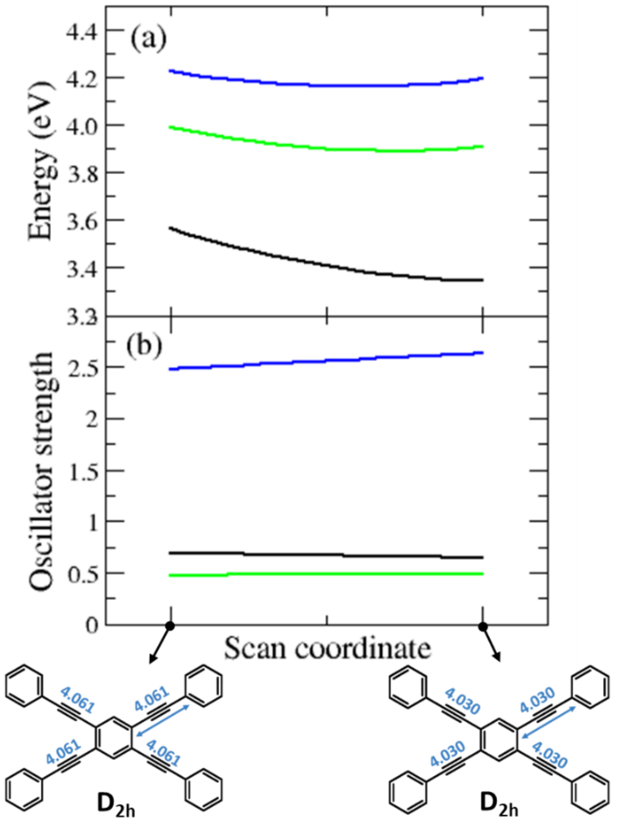

Figure S9. *Results of scan calculations performed on molecule* ***4****. The start and end points of the calculation are reported on the x-axis, corresponding to the optimized ground state geometry and the optimized excited state geometry, respectively. The scan is performed by defining an effective coordinate connecting the ground state geometry,* x_{gs}*, to the excited state geometry,* x_{es}*. Intermediate geometries are obtained moving the atoms from* x_{gs} *along the displacement vector* $d = x_{es}-x_{gs}$*. Bond lengths are reported in Å.*

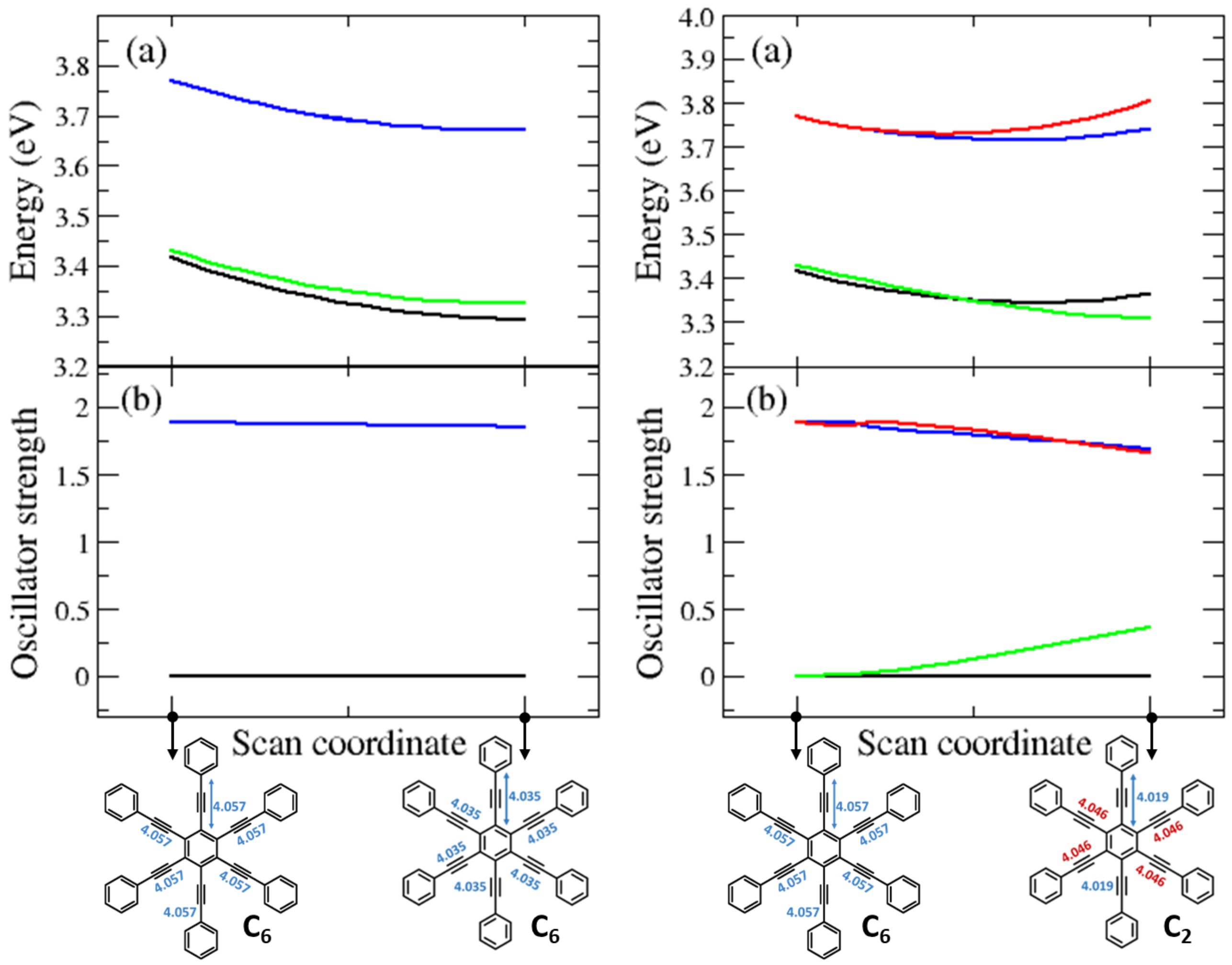

Figure S10. *Results of scan calculations performed on molecule* ***6****. The start and end points of the calculation are reported on the x-axis, corresponding to the optimized ground state geometry and the optimized excited state geometry, respectively. Left panels refer to the scan results from the ground state optimized geometry to the first (vertical) excited state optimized geometry, while right panels refer to the scan results from the ground state optimized geometry to the second (vertical) excited state optimized geometry. The scan is performed by defining an effective coordinate connecting the ground state geometry,* x_{gs}*, to the excited state geometry,* x_{es}*. Intermediate geometries are obtained moving the atoms from* x_{gs} *along the displacement vector* $d = x_{es}-x_{gs}$*. When the molecule is symmetric (left panels), the third and fourth excited states are degenerate. Bond lengths are reported in Å.*

4. ^{1}H and ^{13}C NMR spectra of the PEs

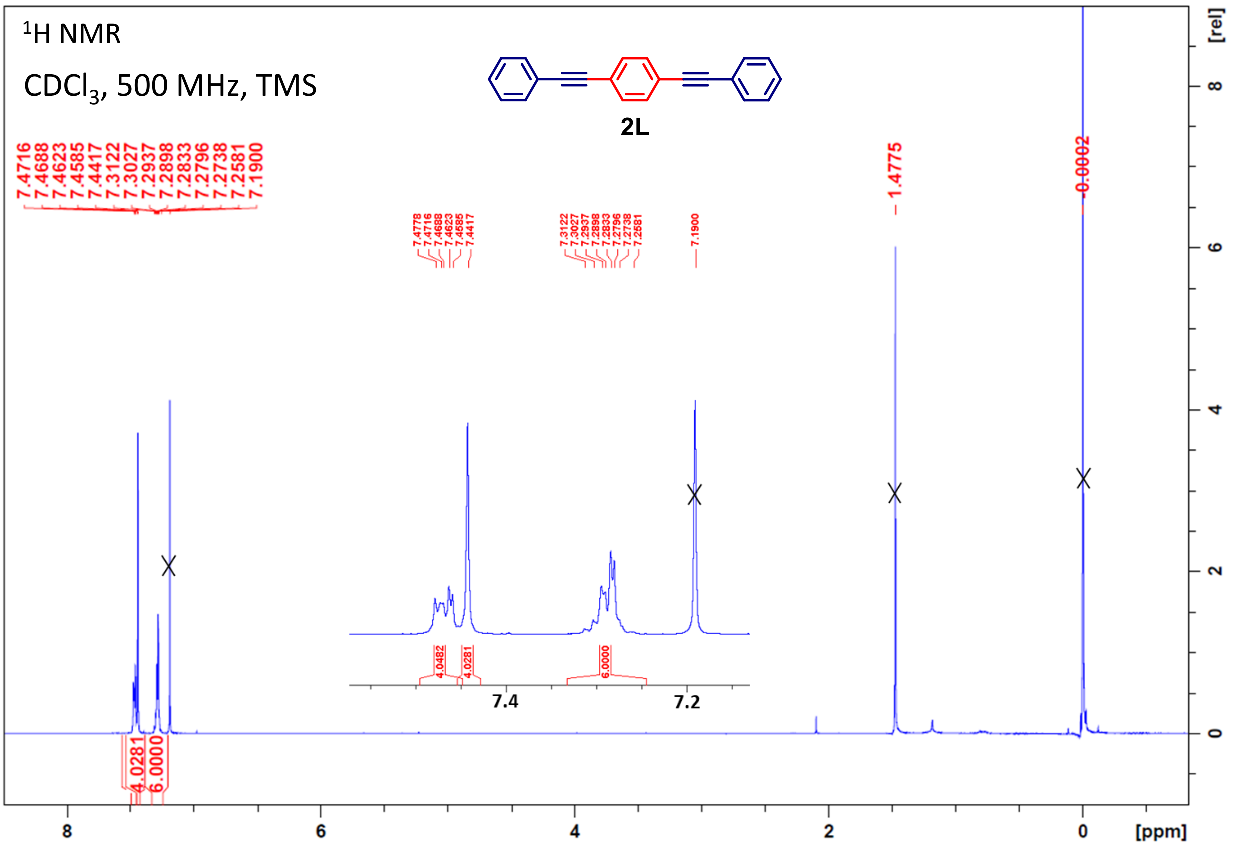

Figure S11: ^{1}H-NMR (500 MHz, 298 K) spectrum of **2L** in chloroform ($CDCl_3$)

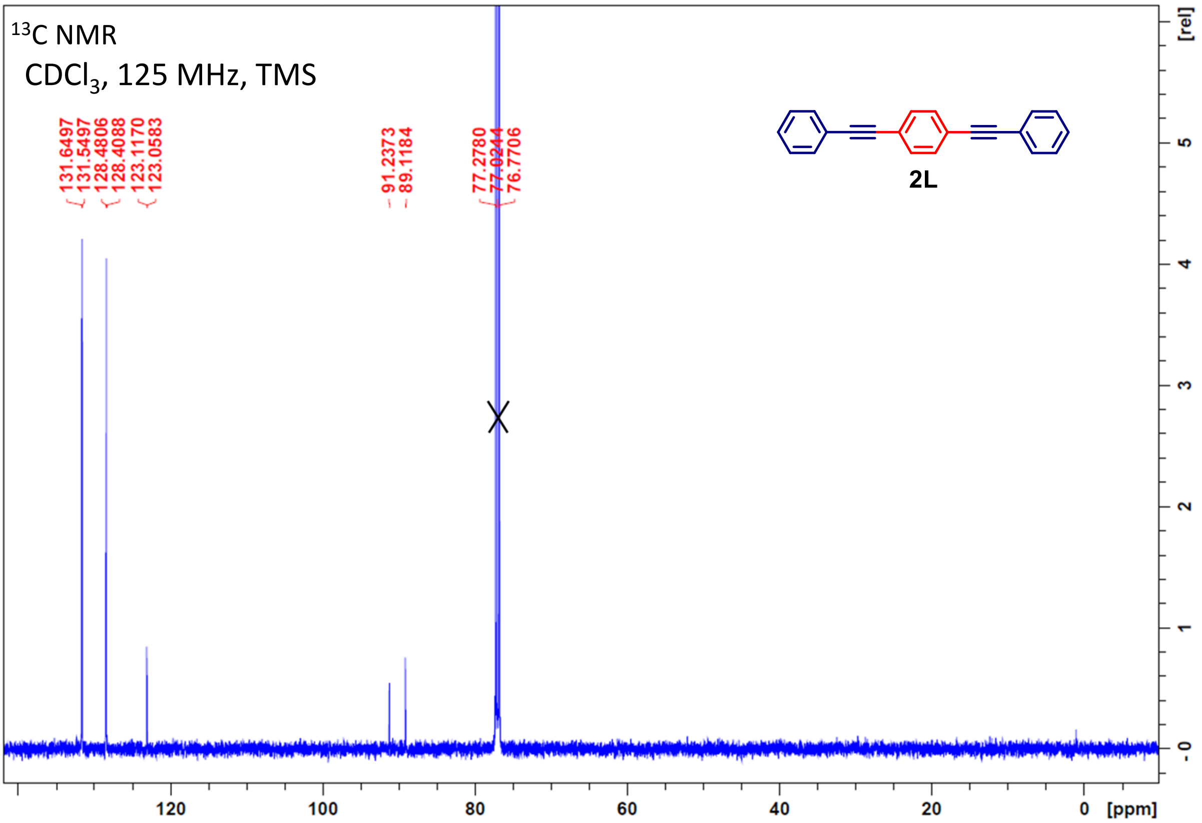

Figure S12: ^{13}C-NMR (125 MHz, 298 K) spectrum of **2L** in chloroform ($CDCl_3$)

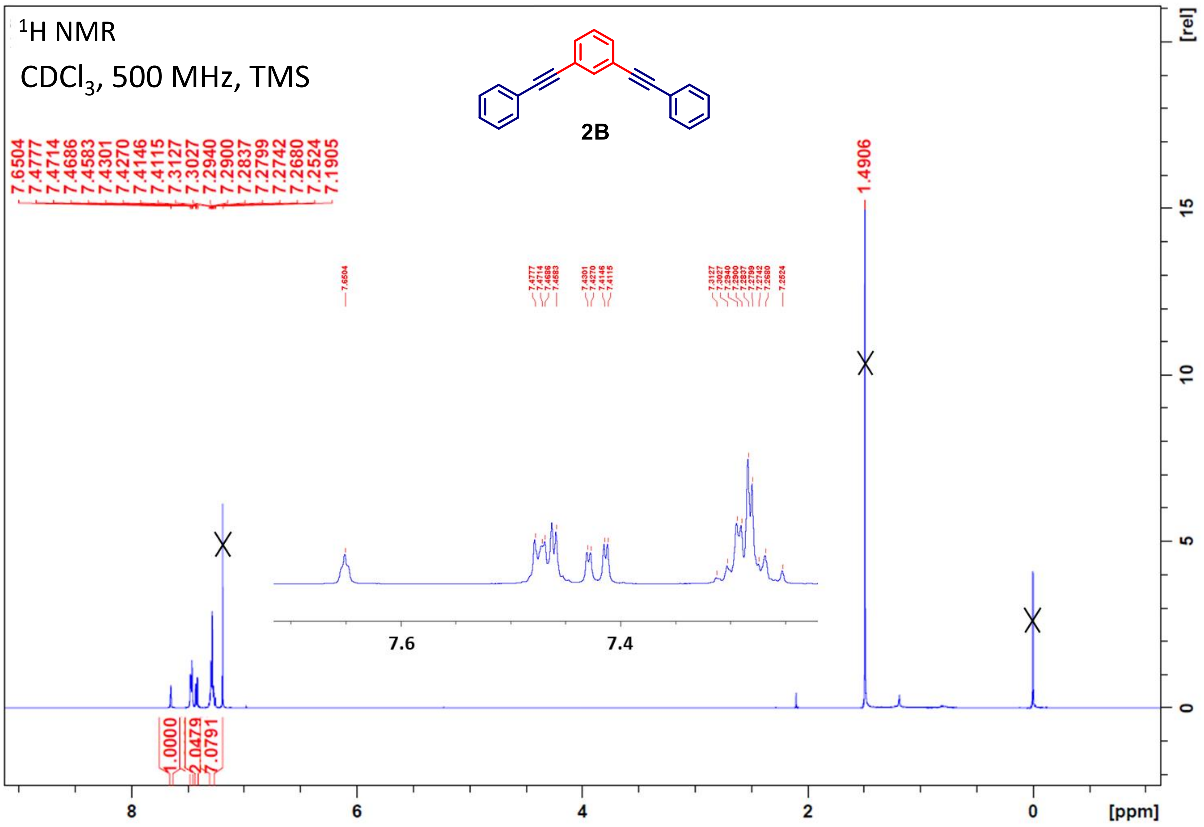

Figure S13: ^{1}H-NMR (500 MHz, 298 K) spectrum of **2B** in chloroform ($CDCl_3$)

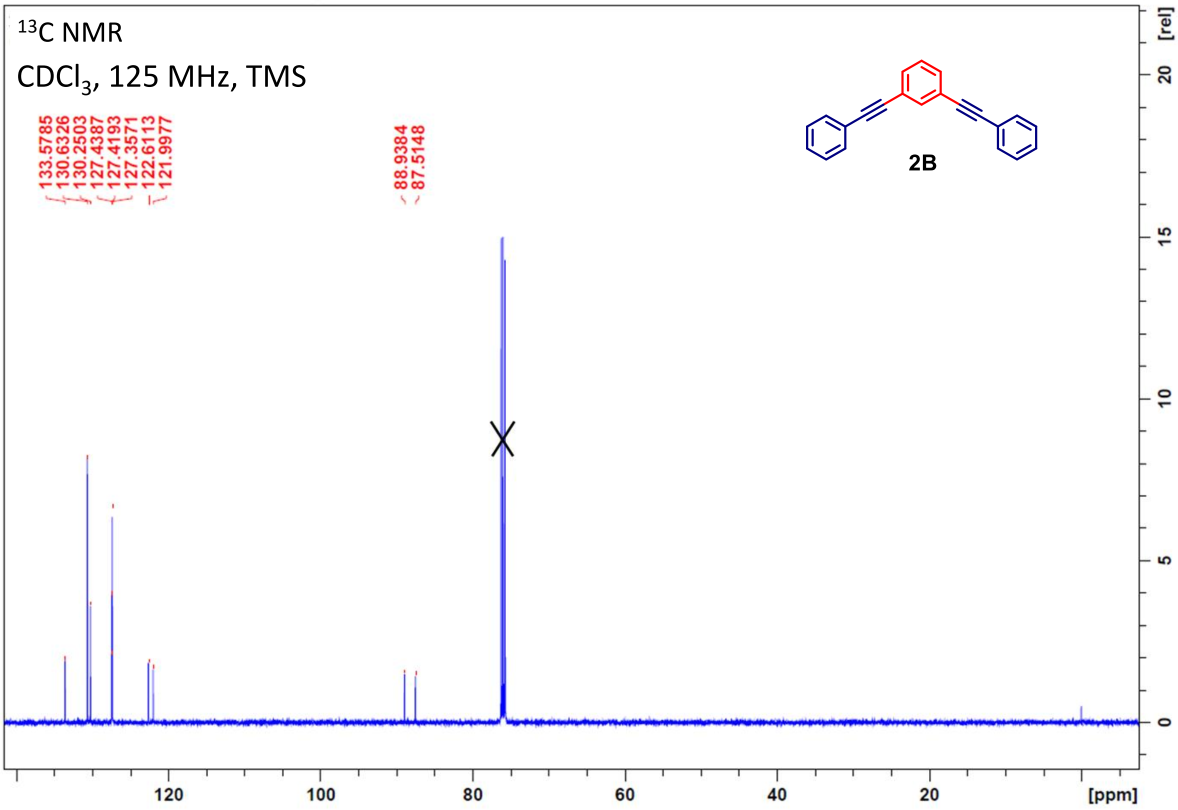

Figure S14: ^{13}C-NMR (125 MHz, 298 K) spectrum of **2B** in chloroform ($CDCl_3$)

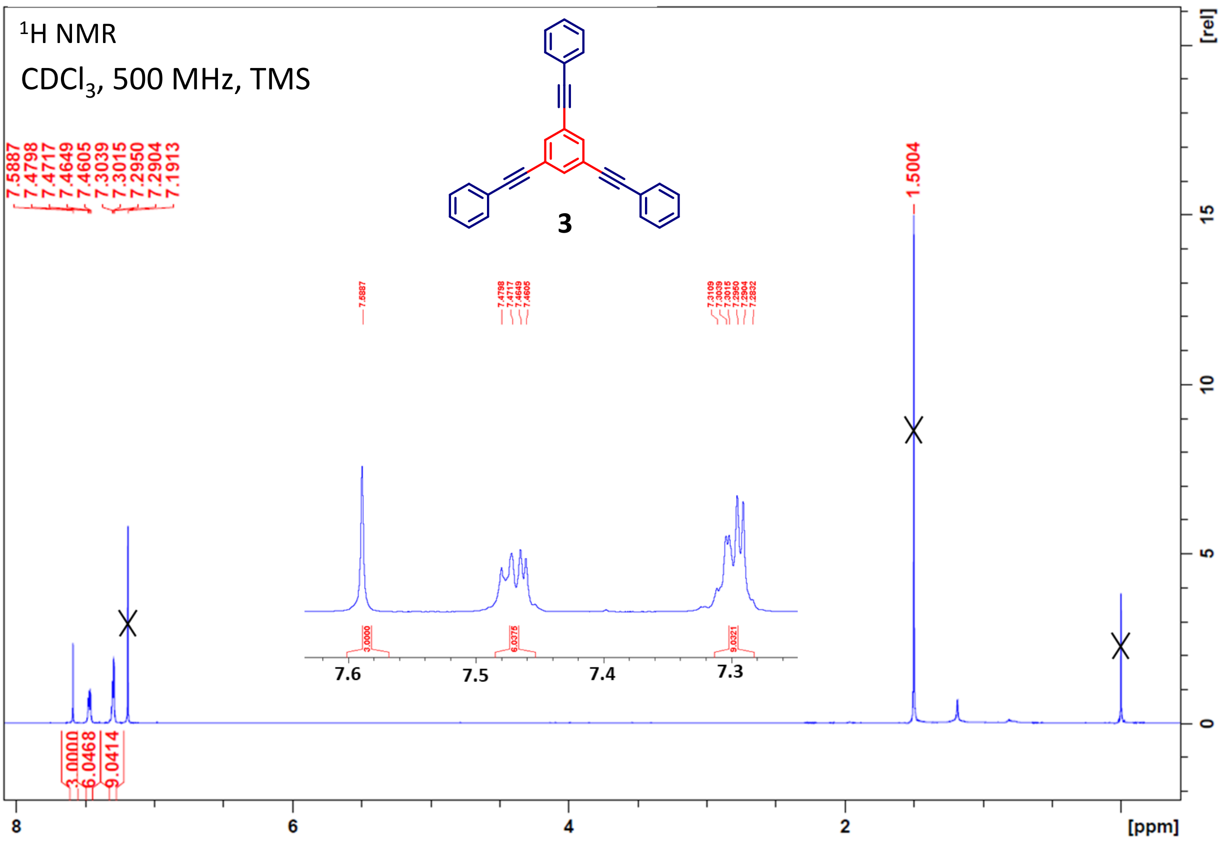

Figure S15: ^{1}H-NMR (500 MHz, 298 K) spectrum of **3** in chloroform ($CDCl_3$)

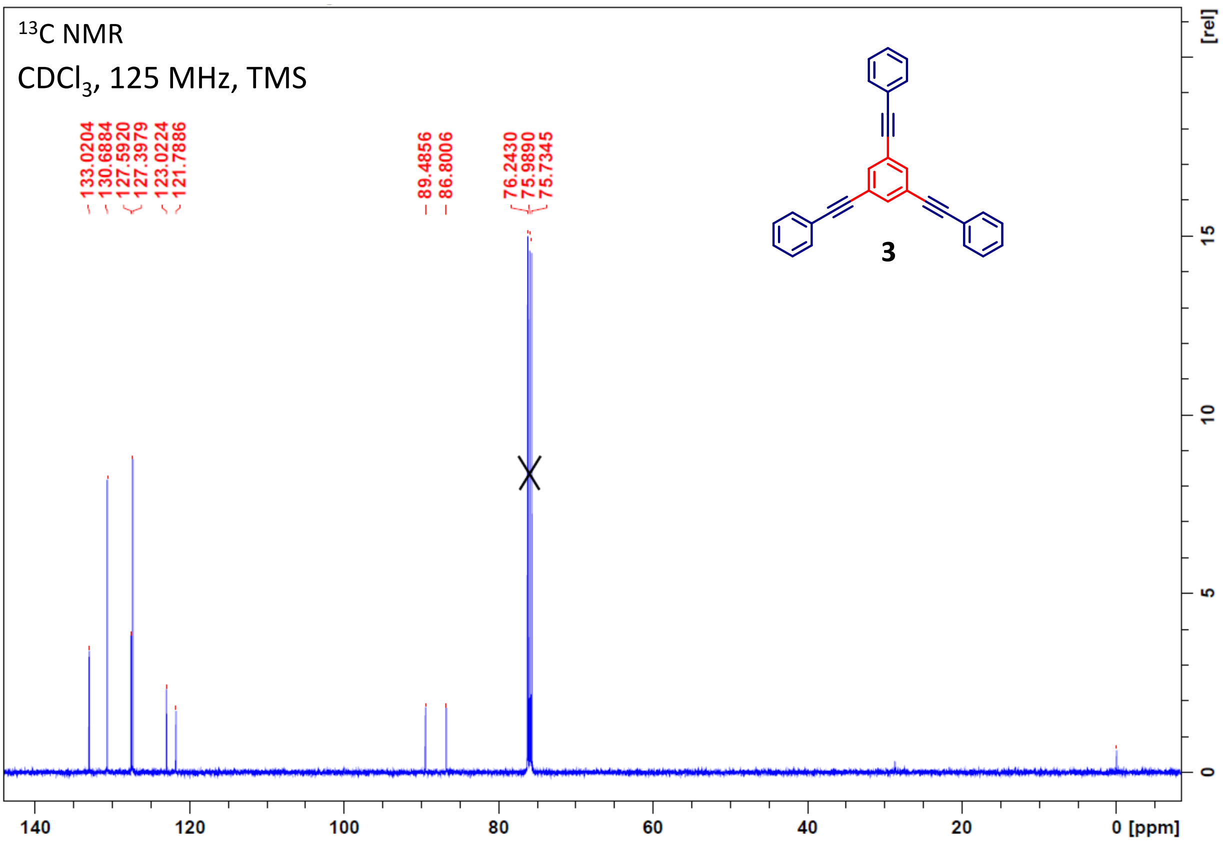

Figure S16: ^{13}C-NMR (125 MHz, 298 K) spectrum of **3** in chloroform ($CDCl_3$)

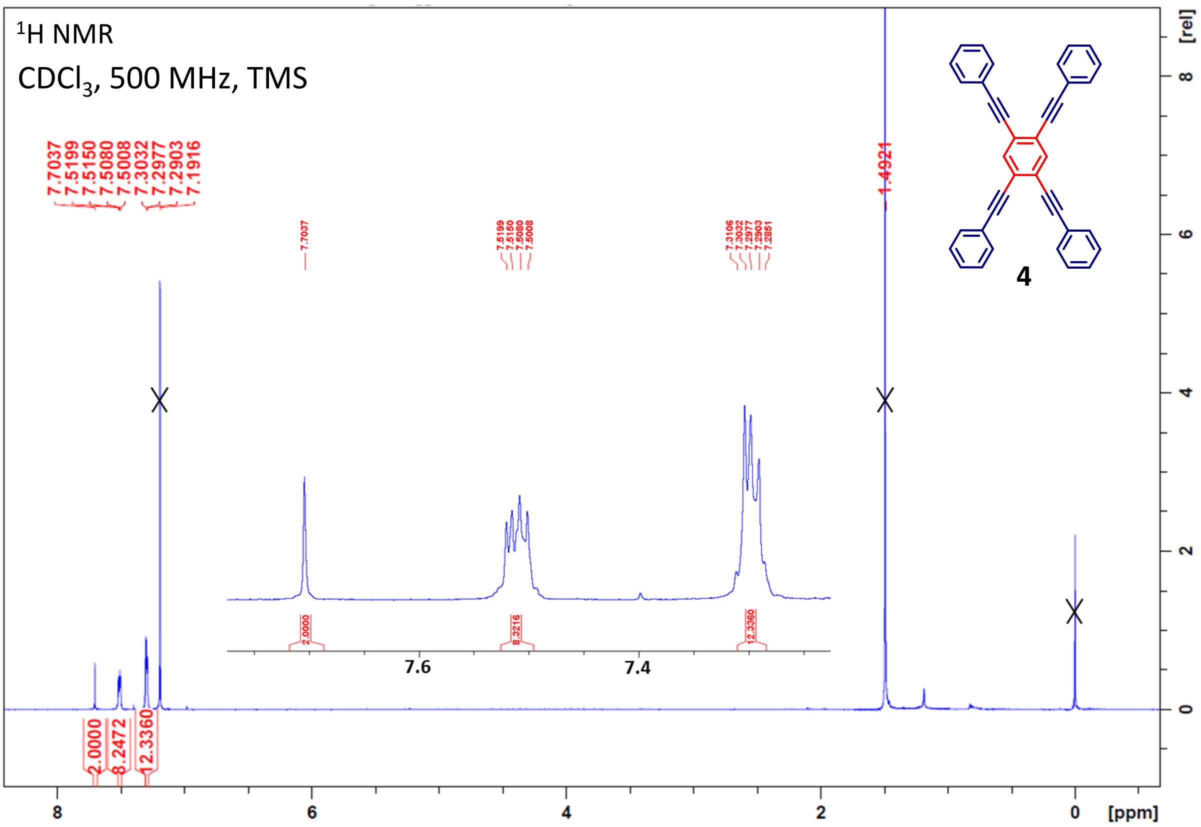

Figure S17: ^{1}H-NMR (500 MHz, 298 K) spectrum of **4** in chloroform ($CDCl_3$)

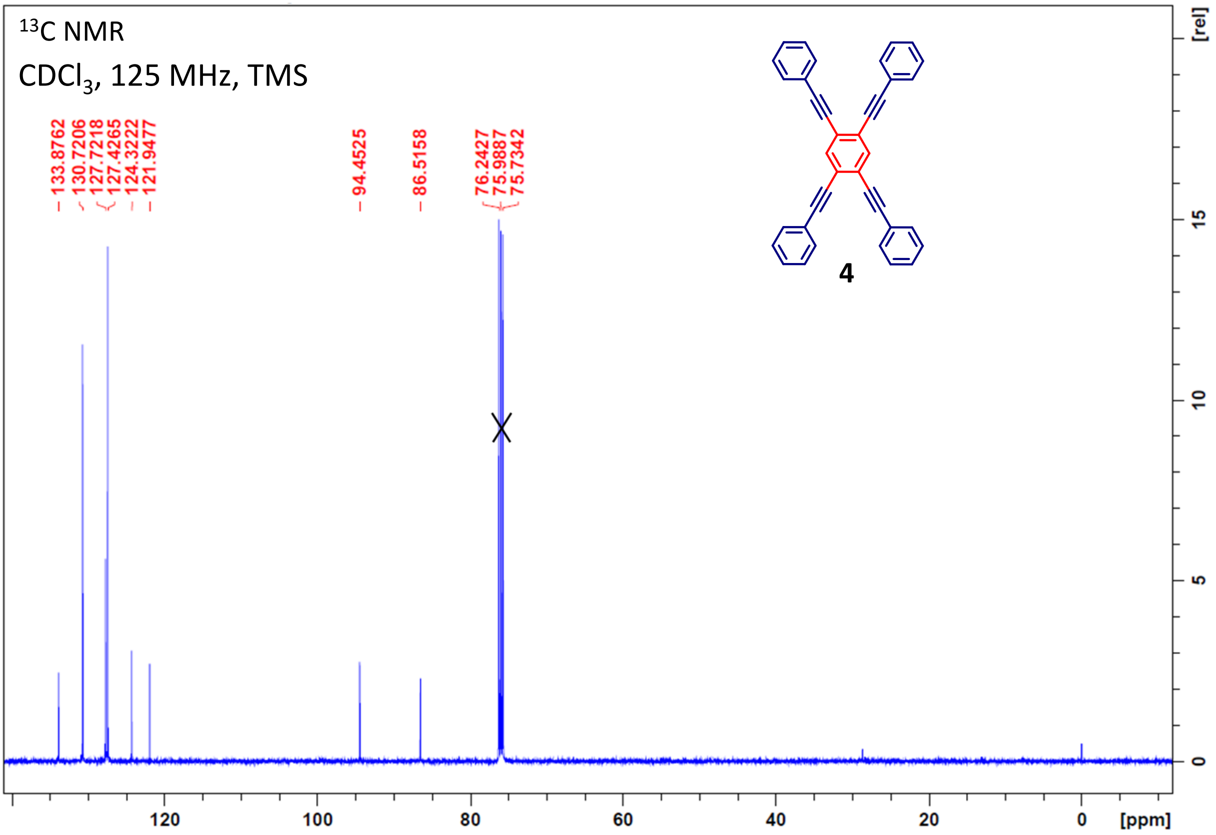

Figure S18: ^{13}C-NMR (125 MHz, 298 K) spectrum of **4** in chloroform ($CDCl_3$)

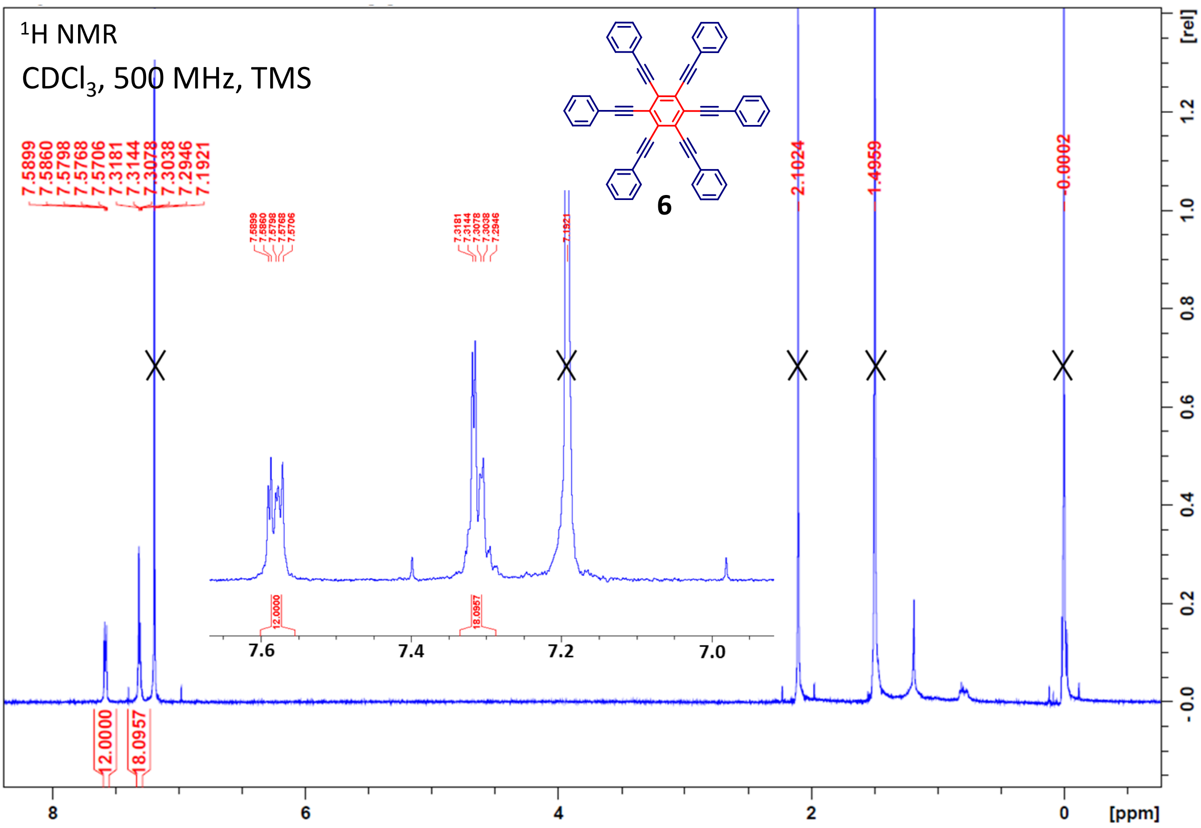

Figure S19: ^{1}H-NMR (500 MHz, 298 K) spectrum of **6** in chloroform ($CDCl_3$)

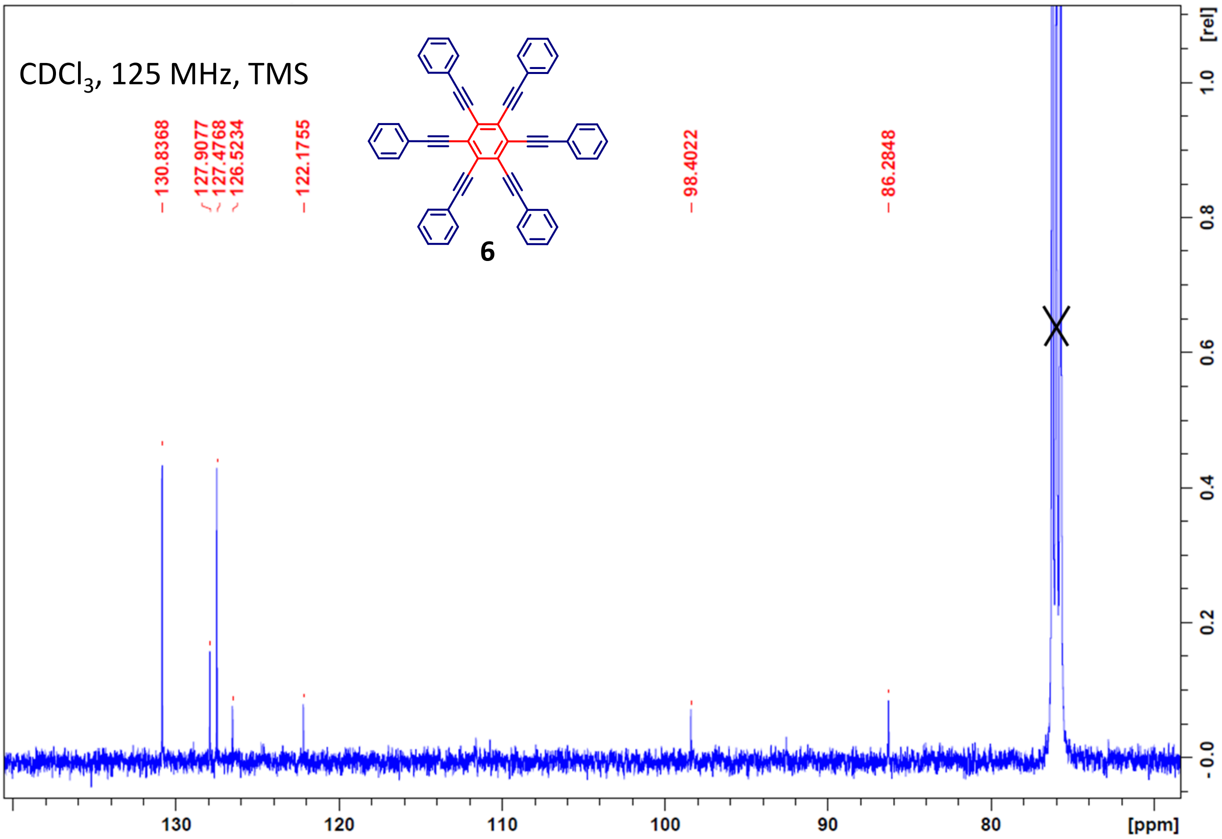

Figure S20: ^{13}C-NMR (125 MHz, 298 K) spectrum of **6** in chloroform ($CDCl_3$)

5. References

1. D. Zimdars, R. S. Francis, C. Ferrante and M. D. Fayer, *J. Chem. Phys.*, 1997, **106**, 7498-7511.
2. J. R. Lakowicz, *Principles of Fluorescence Spectroscopy*, Springer, 2006.
